# Numerical impedance and modulus spectroscopy of CuInSe_2_ thin-film solar cells: relaxation processes and equivalent-circuit analysis

**DOI:** 10.1039/d6ra09020f

**Published:** 2026-11-02

**Authors:** Abdelhadi Mouhiha, Abdelaziz Amine, Reddad El Moznine

**Affiliations:** a Laboratory Physics of Condensed Matter (LPMC), University Chouaib Doukkali El-Jadida Morocco mouhiha.abdelhadi@ucd.ac.ma elmoznine@yahoo.fr

## Abstract

Most numerical studies of CuInSe_2_ (CIS) solar cells rely mainly on current–voltage analysis, while their frequency-dependent electrical processes remain less explored. In this work, an ITO/ZnSe/CuInSe_2_/WSe_2_/Cu-doped C solar cell was investigated using the DC and small-signal AC capabilities of SCAPS-1D. The effects of CIS thickness, series resistance (*R*_s_), shunt resistance (*R*_sh_), and interface defects were systematically examined. An optimal absorber thickness of 1.2 µm yielded a maximum simulated PCE of approximately 33.4%. Increasing *R*_s_ mainly reduced FF and PCE, whereas increasing *R*_sh_ suppressed leakage losses and improved device performance. Interface defects enhanced recombination and mainly affected the low- and intermediate-frequency responses. Combined impedance Z*(*ω*) and modulus M*(*ω*) analyses over 10^−3^–100 MHz revealed three overlapping relaxation contributions. A three-branch equivalent-circuit model was used to separate these contributions and determine their characteristic time constants. Their sensitivities to thickness, parasitic resistances, and interface defects support their tentative association with recombination and leakage, carrier transport, and fast interfacial relaxation. The analysis of the fitted resistances and capacitances clarified the evolution of the relaxation times and provided complementary insight into the simulated photovoltaic response.

## Abstract 2

SCAPS-1D simulations combine photovoltaic, impedance and modulus analyses to examine thickness effects and overlapping electrical relaxation contributions in CIS solar cells.

## Graphical abstract

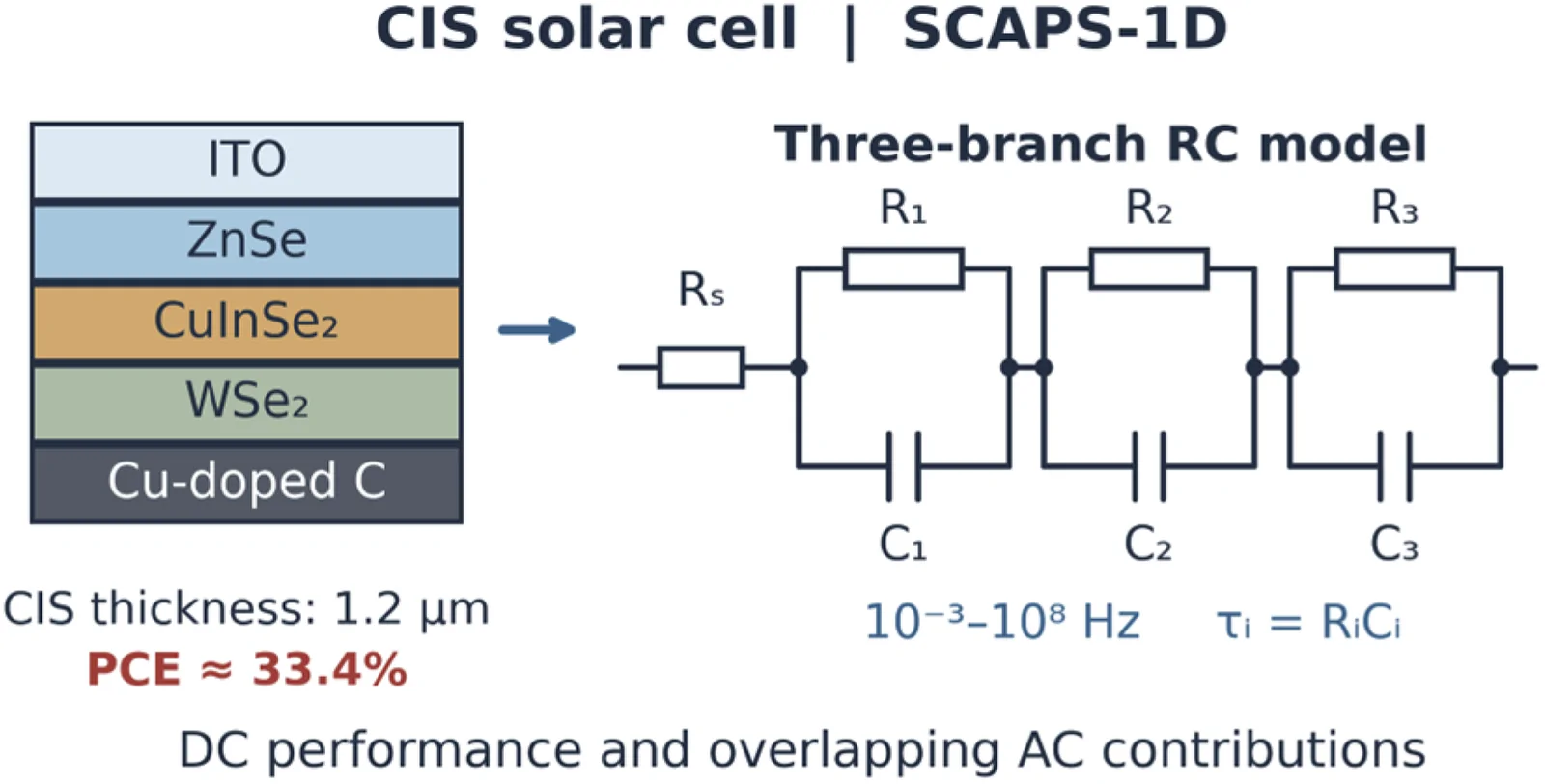

## 1 Introduction

As society continues to advance, energy consumption is steadily increasing. Electrical energy, the most widely utilized form of energy, plays a central role in nearly all aspects of modern life. In light of growing environmental concerns, the development of clean and non-polluting methods for generating electricity has become critically important. Unlike finite and non-renewable resources such as coal, oil, and natural gas, solar energy offers a compelling alternative due to its inexhaustibility, environmental cleanliness, and absence of harmful emissions. As global energy demand escalates, the significance of renewable energy sources—particularly solar power—is increasingly evident, both for their ecological benefits and their potential to reduce dependence on fossil fuels.^1^ Among the various photovoltaic technologies, thin-film copper indium diselenide (CuInSe_2_, CIS) has emerged as one of the most promising absorber materials due to its optimal direct band gap (∼1.0 eV), high absorption coefficient (>10^5^ cm^−1^), and favorable optoelectronic properties.^2,3^ These features make CIS suitable for high-efficiency and stable solar cells. To design and optimize such devices, numerical simulation tools play a crucial role. SCAPS-1D is widely used to model the electrical behavior of thin-film solar cells under steady-state (DC) conditions, providing valuable insights into the effect of structural and electronic parameters. In particular, parameters such as absorber thickness, shunt resistance (*R*_sh_), series resistance (*R*_s_), and interface defect density are critical, as they affect carrier collection, recombination, and electrical losses, thereby influencing the power conversion efficiency (PCE).

More recently, Riaz *et al.*^4^ investigated an ITO/ZnSe/CuInSe_2_/WSe_2_/Au solar cell using the DC and AC capabilities of SCAPS-1D. Their study included photovoltaic characterization, thickness- and temperature-dependent impedance analysis, electric-modulus spectra, and equivalent-circuit fitting. Their equivalent-circuit analysis described the response using a single RC relaxation. This raises the question of whether such a description remains sufficient over an extended frequency range, particularly when weak contributions are masked by the dominant impedance response. The present study examines this question and investigates how absorber thickness, external resistances, and interface defect states affect the spectral features and fitted relaxation parameters.

Impedance spectra may contain overlapping contributions from recombination, leakage pathways, carrier transport, charge accumulation, and interfaces. Two contributions with similar characteristic times can appear as a single peak or arc, while a weaker contribution may be masked by a stronger one. The electric modulus M*(*ω*) provides a complementary representation of the same AC response and may emphasize features that are weak in the complex impedance Z*(*ω*). Previous studies of CIGS solar cells have used multiple RC elements to describe contributions from the junction, buffer layer, and non-ohmic contacts.^5,6^ Equivalent-circuit decomposition can therefore help examine overlapping contributions and estimate their effective resistances, capacitances, and characteristic times. However, the need for additional components must be assessed by comparing alternative models, and their physical interpretation requires supporting evidence from controlled variations of device parameters.

Building on the work of Riaz *et al.*,^4^ this study investigates an ITO/ZnSe/CuInSe_2_/WSe_2_/Cu-doped C solar cell using the DC and small-signal AC capabilities of SCAPS-1D. The Cu-doped carbon rear contact was selected as a potentially lower-cost alternative to the Au electrode used in the reference device, motivated in part by the cost considerations reported by Li *et al.*^5^ Its use in previous numerical studies^6,7^ and the experimental incorporation of Cu-doped graphite into a thin-film photovoltaic rear-contact structure^8^ provide further support for this choice.

The effects of CIS absorber thickness, series resistance, shunt resistance, and absorber-related interface defect states are examined through current–voltage characteristics, complex impedance, and electric modulus. The AC analysis covers a frequency range from 10^−3^ to 10^8^ Hz. A three-branch RC equivalent circuit is used to describe overlapping spectral contributions and extract their characteristic time constants. Finally, the evolution of these time constants is compared with the PCE to explore possible associations with leakage and recombination, carrier transport, and fast interfacial relaxation. These associations are discussed as tentative physical interpretations rather than mechanisms established by correlation alone.

## 2 Methodology and device structure

The simulations in this study were performed using SCAPS-1D (Solar Cell Capacitance Simulator),^9^ a software developed for the numerical analysis of multilayer solar cells. The program allows the physical properties of each layer, including thickness, band gap, electron affinity, dielectric permittivity, carrier mobility, doping density, and defect density, to be defined. The operating conditions and the electrical properties of the front and back contacts can also be specified. Both DC and small-signal AC simulations were performed. The current–voltage characteristics were used to determine the short-circuit current density (*J*_sc_), open-circuit voltage (*V*_oc_), fill factor (FF), and power conversion efficiency (PCE). The AC response was calculated at a DC bias of 0 V over a frequency range from 10^−3^ to 10^8^ Hz. Unless otherwise specified, the series and shunt resistances were fixed at 0.5 Ω cm^2^ and 10^4^ Ω cm^2^, respectively. These values were used as the reference conditions for the absorber-thickness and interface-defect analyses. During the resistance studies, *R*_s_ and *R*_sh_ were varied separately while all other simulation parameters were kept constant. The investigated solar cell followed the ITO/ZnSe/CuInSe_2_/WSe_2_/Cu-doped C configuration shown in Fig. 1(a). The ITO layer deposited on glass served as the transparent front contact, while ZnSe acted as the electron transport layer. CuInSe_2_ a chalcopyrite semiconductor, was used as the absorber, WSe_2_ served as the hole transport layer, while Cu-doped carbon was represented by a rear contact with a work function of 5.0 eV.^10^

**Fig. 1:**
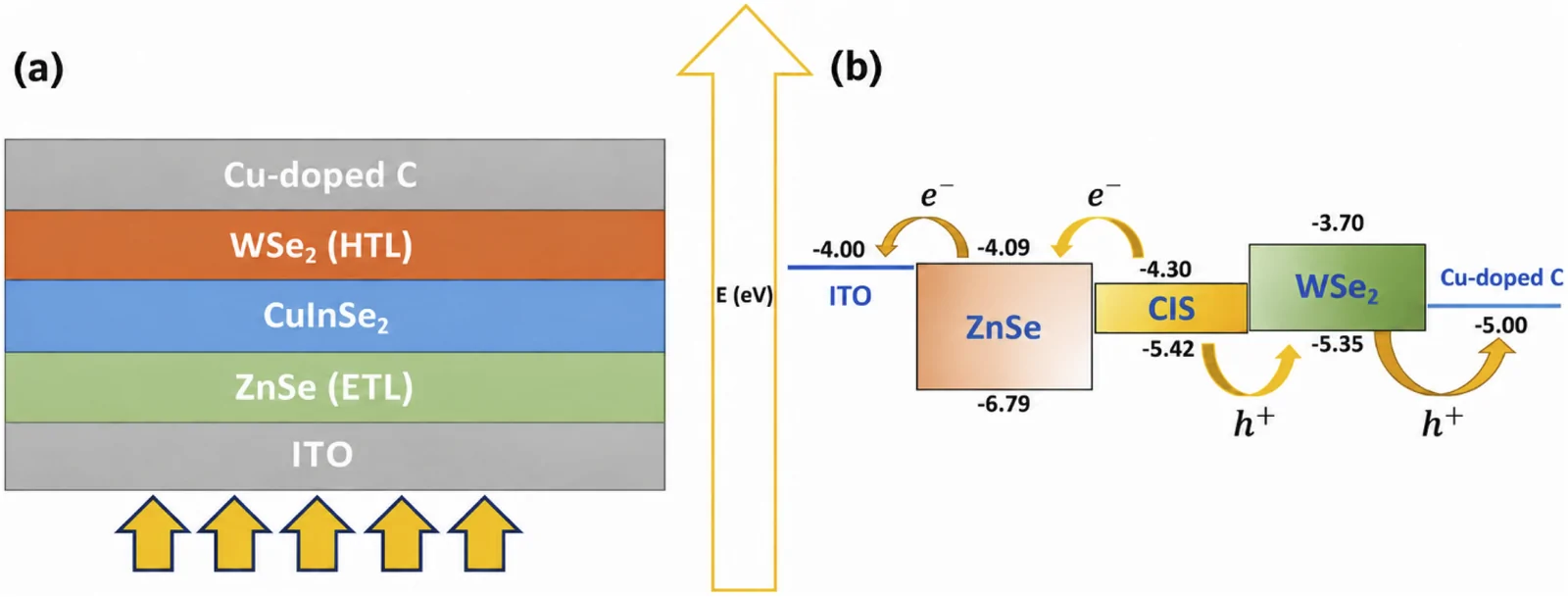
(a) Schematic structure of solar cell and (b) energy diagram.

The physical parameters used for each layer are summarized in Table 1, while the interface-defect properties are presented in Table 2. All simulations were performed at 300 K under AM1.5G illumination with an incident power density of 1000 W m^−2^.

**Table 1:** The input parameters of different layers

Parameters	ITO^11^	ZnSe^12^	CIS^13^	WSe_2_ (ref. 13)
Thickness (nm)	50	80	200–1600	100
Band gap *E*_g_ (eV)	3.5	2.7	1.12	1.65
Electron affinity *χ* (eV)	4.0	4.09	4.3	3.7
Dielectric permittivity (relative)	9	10	10	13.8
CB effective DOS *N*_n_ (cm^−3^)	2.2 × 10^18^	1.5 × 10^18^	2.2 × 10^18^	8.3 × 10^18^
VB effective DOS *N*_v_ (cm^−3^)	1.8 × 10^19^	1.8 × 10^19^	1.8 × 10^19^	1.6 × 10^19^
Electron mobility *µ*_n_ (cm^2^ V^−1^ s^−1^)	20	50	100	100
Hole mobility *µ*_p_ (cm^2^ V^−1^ s^−1^)	10	20	25	500
Thermal velocity of electrons *v*_th_ (ms^−1^)	1 × 10^7^	1 × 10^7^	1 × 10^7^	1 × 10^7^
Thermal velocity of holes *v*_th_ (ms^−1^)	1 × 10^7^	1 × 10^7^	1 × 10^7^	1 × 10^7^
Donor density *N*_d_ (cm^−3^)	1 × 10^21^	1 × 10^18^	0	0
Acceptor density *N*_a_ (cm^−3^)	0	0	1 × 10^19^	1 × 10^20^
Optimized defect density (cm^−3^)	1 × 10^15^	1 × 10^14^	1 × 10^14^	1 × 10^15^
Capture cross section (electron & hole)	1 × 10^−15^	1 × 10^−15^	1 × 10^−15^	1 × 10^−15^

**Table 2:** Interface defect parameters

Interface	Defect type	Capture cross section electrons/holes (cm^2^)	Energetic distribution	Reference for defect energy level	Total density (cm^−2^)
ETL/absorber	Neutral	1 × 10^−19^/1 × 10^−19^	Single	Above VB_max_ by 0.6 eV	1× 10^12^
Absorber/HTL	Neutral	1 × 10^−19^/1 × 10^−19^	Single	Above VB_max_ by 0.6 eV	1× 10^12^

## 3 Results and discussion

### 3.1 Effect of back-contact work function on photovoltaic performance

The effect of the back-contact work function was examined over the range of 4.9–5.5 eV, keeping all other device parameters and simulation conditions unchanged. This allows the influence of the contact work function to be assessed independently of changes in the layers, interfaces, or resistive losses. The photovoltaic parameters obtained from the illuminated *J*–*V* curves are reported in Table 3. The PCE increased from 33.3836% at 4.9 eV to 33.4373% at 5.0 eV, mainly because the FF increased from 84.4685% to 84.6044%. In comparison, *V*_oc_ and *J*_sc_ changed very little. Above 5.0 eV, increasing the work function produced only small variations in the photovoltaic parameters, indicating an approach to a plateau within the investigated range. The work functions adopted for Cu-doped carbon and Au were 5.0 and 5.1 eV, respectively. These values gave efficiencies of 33.4373% and 33.4384%, corresponding to a relative decrease of only 0.00346% for Cu-doped carbon compared with Au. The FF values were also close, at 84.6044% and 84.6073%, respectively, while *V*_oc_ and *J*_sc_ showed negligible differences. The similar photovoltaic parameters obtained with Au and Cu-doped carbon reflect the weak dependence of device performance on the back-contact work function in this range. Under the adopted simulation conditions, Cu-doped carbon can therefore replace Au with only a marginal change in DC performance.

**Table 3:** Effect of the assumed back-contact work function on the simulated photovoltaic parameters

Back-contact material	Work function (eV)^6,14^	PCE (%)	*V*_oc_ (V)	*J*_sc_ (mA cm^−2^)	FF (%)
Mo(110)	4.93	33.38358	0.924398	42.75423	84.46851
Cu-doped carbon	5.0	33.43727	0.924395	42.75432	84.60440
Au	5.1	33.43842	0.924395	42.75432	84.60732
W	5.2	33.43845	0.924395	42.75432	84.60739
Ni	5.5	33.43864	0.924396	42.75456	84.60737

### 3.2 Current–voltage characteristics effect of the active layer thickness

Fig. 2(a and b) presents the illuminated *J*–*V* characteristics of the proposed CIS solar cell for different absorber thicknesses. Increasing the thickness produces a pronounced increase in *J*_sc_, accompanied by a smaller decrease in *V*_oc_. The corresponding QE spectra, shown in Fig. 2(c), help explain the increase in photocurrent. For the 0.2 µm absorber, QE decreases markedly toward longer wavelengths, indicating a limited contribution of these photons to the collected current. Increasing the CIS thickness extends the region of high QE toward the absorption edge, consistent with improved absorption of weakly absorbed photons. The spectra become progressively closer over the visible region, while differences remain more apparent near the absorption edge. In particular, the 1.6 µm absorber maintains a higher long-wavelength response than the 1.2 µm absorber. The cutoff wavelength remains nearly unchanged, consistent with the fixed CIS band gap.

**Fig. 2:**
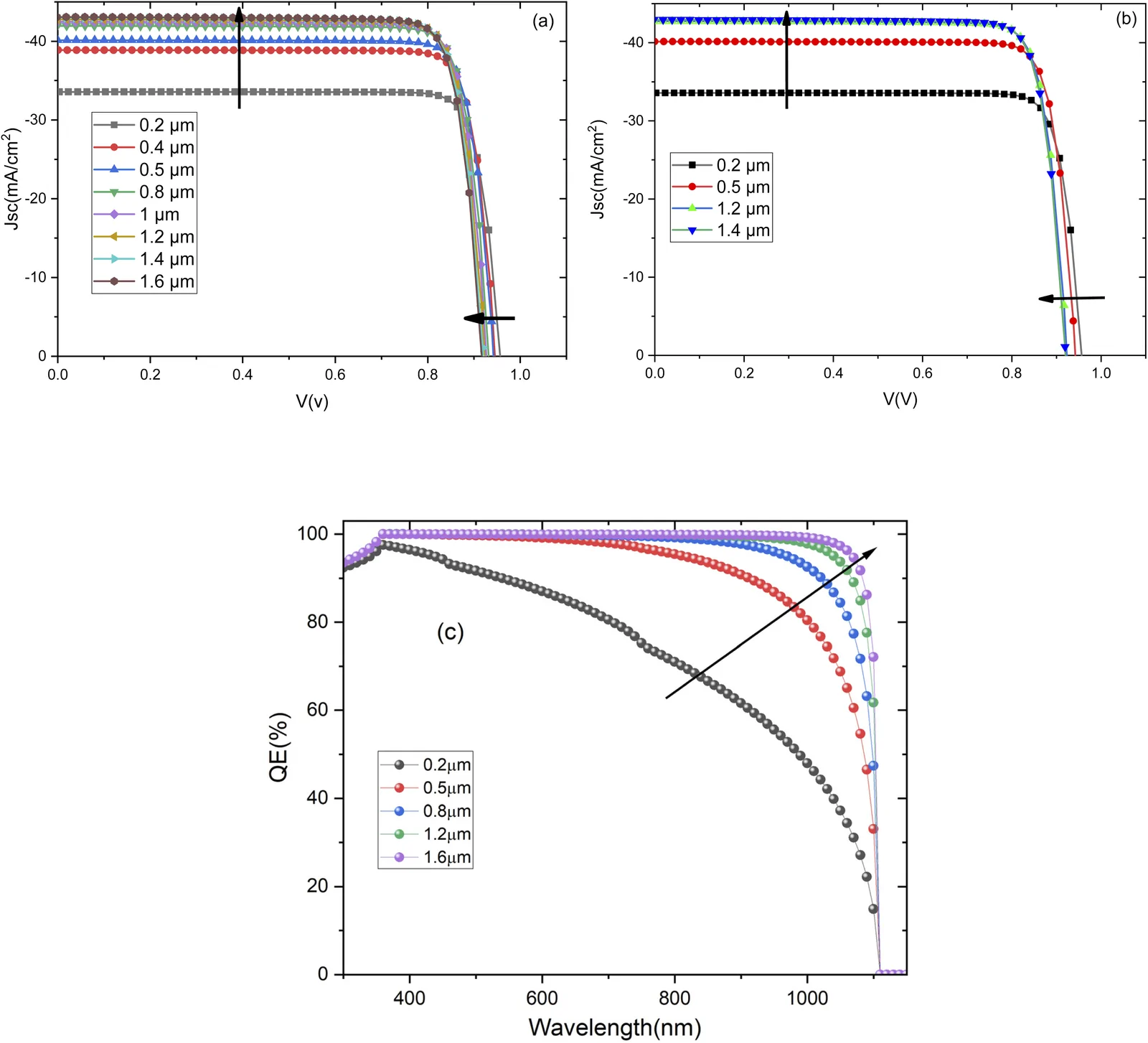
(a and b) Illuminated *J*–*V* characteristics of the proposed CIS solar cell for different absorber thicknesses; (c) corresponding QE spectra for selected thicknesses.

The absorber layer thickness influences solar cell performance by governing photon absorption and charge carrier generation. An excessively thin layer limits the capture of incident photons, thereby reducing carrier production,^15^ whereas a thick absorber can hinder charge transport and collection when its thickness becomes comparable to or exceeds the relevant carrier diffusion length.^16^ In this study, the CIS absorber thickness was varied from 0.2 to 1.6 µm while all other simulation parameters were kept unchanged. Fig. 3 summarizes the resulting photovoltaic parameters. As the CIS thickness increases, we note a significant improvement in the short-circuit current density (*J*_sc_), for instance, rising from approximately 33.5 mA cm^−2^ at 0.2 µm to over 42.5 mA cm^−2^ at 1.6 µm. This increase is consistent with the improved long-wavelength QE shown in Fig. 2(c). However, this increase in thickness is accompanying with a gradual decrease in the open-circuit voltage (*V*_oc_), which declines from around 0.955 V at 0.2 µm to approximately 0.918 V at 1.6 µm. This trend may be associated with increased recombination losses as thickness grows, as discussed in previous studies.^17–19^ Concurrently, the fill factor (FF) also exhibits a continuous decrease, falling from about 85.7% at 0.2 µm to nearly 84.2% at 1.6 µm, which may reflect thickness-dependent transport and recombination losses.^20^ Consequently, the overall cell efficiency (PCE), resulting from the interplay of these behaviors, presents a non-monotonic curve. It experiences an initial phase of growth, primarily driven by the increase in *J*_sc_, reaching its highest value among the investigated thicknesses, approximately 33.4%, at 1.2 µm. Beyond this thickness, despite substantial light absorption, the continuing decrease of *V*_oc_ and FF leads to a slight reduction in efficiency. Further increases in thickness provide diminishing benefits for PCE, although the QE continues to improve near the absorption edge. Additional transport and recombination losses may contribute to this behavior, as discussed in other thin-film photovoltaic systems.^4,21,22^ These observations highlight the importance of optimizing the absorber thickness to balance the photocurrent gain against the accompanying decreases in *V*_oc_ and FF, thereby maximizing the simulated photovoltaic efficiency. These mechanisms are proposed as possible explanations; their individual contributions cannot be distinguished from the *J*–*V* and QE results alone.

**Fig. 3:**
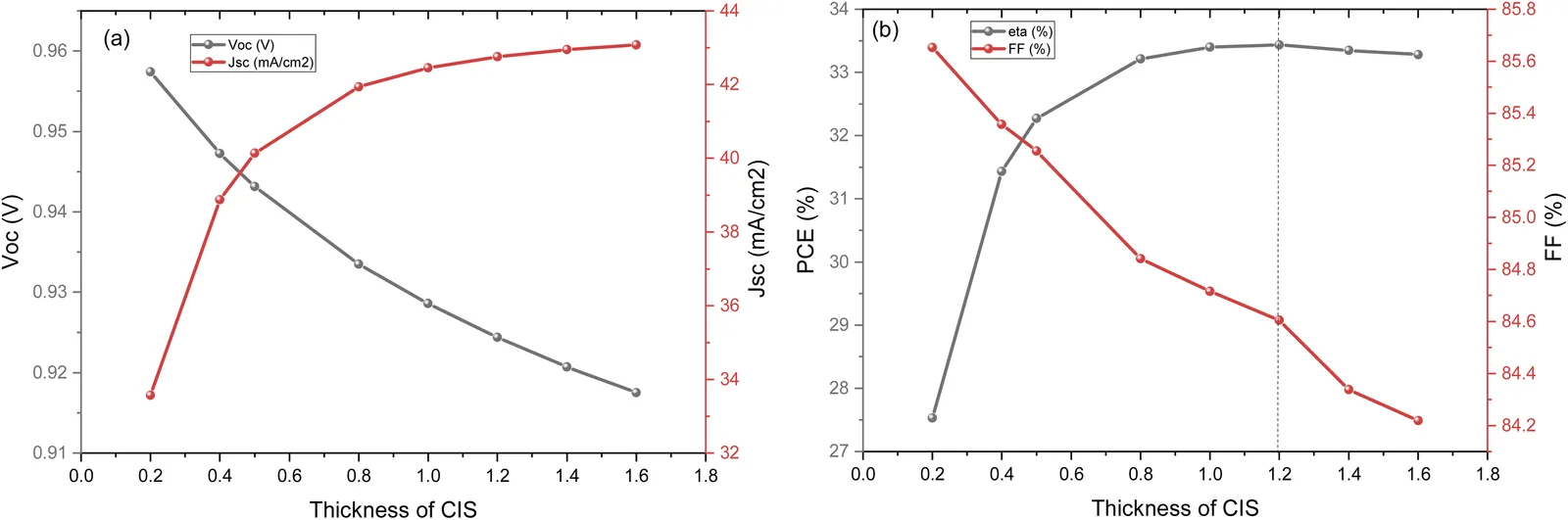
Effect of absorber layer thickness on (a) *V*_oc_, *J*_sc,_ (b) FF and PCE.

### 3.3 Influence of series resistance (*R*_s_) on solar cell performance

The performance of chalcopyrite-based solar cells is notably influenced by the series resistance (*R*_s_), which acts as a parasitic component in the current path. To evaluate its effect, simulations were carried out at the optimized absorber thickness of 1.2 µm while varying *R*_s_ from 0 to 1 Ω cm^2^. As shown in Fig. 4(a), increasing *R*_s_ leads to a continuous reduction in both the fill factor (FF) and the power conversion efficiency (PCE). Specifically, FF decreases from 87% to 82%, while PCE drops from approximately 34.4% to 32.6%. These reductions are consistent with increased ohmic losses when the device delivers current.^23^Fig. 4(b) shows that *J*_sc_ remains nearly unchanged over the investigated *R*_s_ range, while the extracted *V*_oc_ exhibits a slight increase. The voltage drops across the lumped external series resistance is given by Δ*V*_*R*_s__ = *JR*_s_ where *J* is the terminal current density. At open circuit, *J* = 0, so this voltage drop is zero. Increasing *R*_s_ alone is therefore not expected to change *V*_oc_ when all other model parameters remain fixed. The slight increase in the extracted *V*_oc_ may arise from numerical effects and is not interpreted as a physical consequence of increasing *R*_s_. Similar slight variations have been reported in SCAPS-1D simulations and discussed as potentially numerical.^24^ The dominant effect of *R*_s_ in the investigated range is thus the reduction Sin FF and PCE, with comparatively little variation in *J*_sc_ and *V*_oc_.

**Fig. 4:**
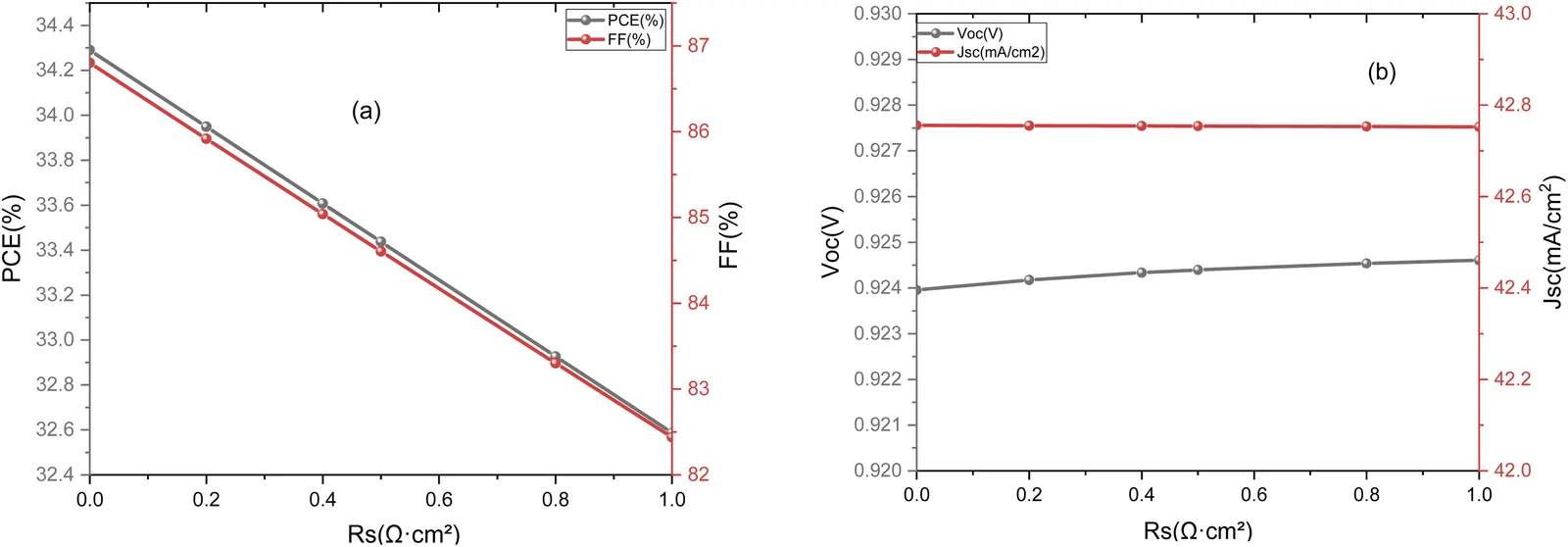
Effect of *R*_s_ on (a) FF, PCE and (a) *V*_oc_, *J*_sc_.

### 3.4 Influence of shunt resistance (*R*_sh_) on solar cell performance

The shunt resistance (*R*_sh_) is a critical parameter that significantly impacts solar cell performance Shunt resistance (*R*_sh_) represents parasitic current leakage pathways in parallel with the active device. Such pathways can be associated with fabrication defects or non-ideal interfaces.^25^ To specifically highlight the effect of *R*_sh_ a simulation was carried out by varying *R*_sh_ from 10^2^ to 10^11^ ohm cm^2^. To accurately isolate its influence from series losses, the value of series resistance (*R*_s_) was kept at 0 Ω cm^2^ throughout the evaluations. Fig. 5(a) and (b) illustrate the direct correlation between shunt resistance and key solar cell optoelectrical parameters. As shunt resistance increases, the photoconversion efficiency (PCE) shows a significant improvement, increase from roughly 27% and saturating around 34%. Similarly, the fill factor (FF) increases dramatically from about 68% and saturates near 87% over the same *R*_sh_ range. These strong initial dependencies highlight that very low *R*_sh_ values severely degrade the cell's ability to deliver power, primarily because current bypasses the load through leakage paths, thus reducing the power output and filling of the *J*–*V* curve. The open-circuit voltage (*V*_oc_) also exhibits an improvement with increasing shunt resistance, rising from approximately 0.918 V at lower *R*_sh_ values to around 0.924 V before saturation. This indicates that a very low *R*_sh_ allows a significant leakage current even at open-circuit conditions, reducing the maximum voltage the cell can maintain. In contrast, the short-circuit current density (*J*_sc_) remains relatively stable. These trends suggest that a shunt resistance more than 10^4^ Ω cm^2^, is crucial for enhancing the solar cell's efficiency, fill factor, and open-circuit voltage, thereby improving its overall performance. These observations are consistent with the understanding that increased leakage at low *R*_sh_ severely limits performance, while sufficiently high *R*_sh_ minimizes these losses, allowing the intrinsic properties of the cell to dominate. Several works have reported similar PV performances influenced by shunt resistance.^26–29^

**Fig. 5:**
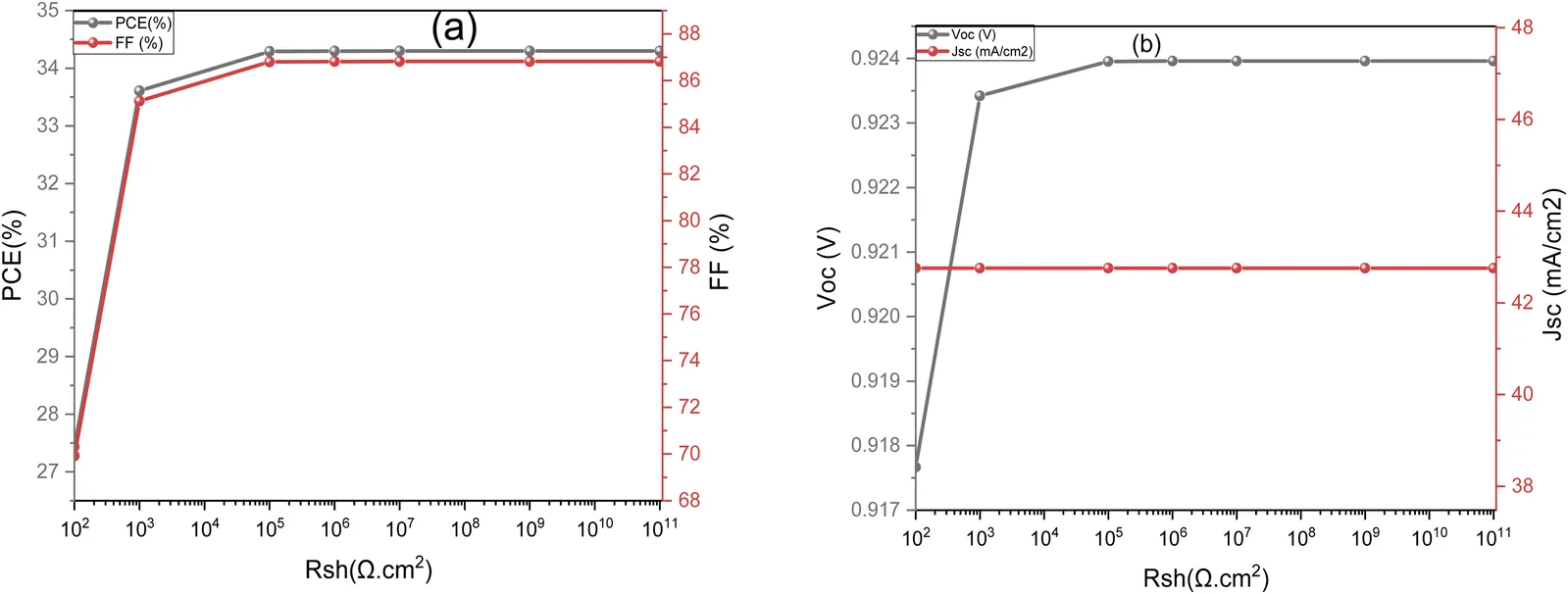
Effect of *R*_sh_ on (a) *V*_oc_, *J*_sc_, (b) FF and PCE.

### 3.5 Electric field, energy bands, and carrier transport

To examine the relationship between the internal electric field and carrier transport, Fig. 6 presents the electric field, energy bands, current densities, and carrier concentrations of the Cu-doped C/WSe_2_/CIS/ZnSe/ITO structure. These profiles were calculated under illumination at an applied bias of approximately 0 V. Fig. 6(a) shows that the electric field is concentrated near the contacts and interfaces, while most of the CIS absorber remains nearly field-free. A strong positive field occurs near the Cu-doped C/WSe_2_ contact and decreases rapidly within WSe_2_. With the spatial coordinate increasing toward ITO, this field opposes hole drift toward the rear electrode. Negative field regions are observed near CIS/ZnSe and ZnSe/ITO, favoring electron drift toward the front contact. The largest field magnitude occurs near ZnSe/ITO. The energy-band profiles in Fig. 6(b) are consistent with this field distribution. The conduction and valence bands remain nearly flat throughout the central CIS region, whereas pronounced bending occurs near the contacts. The discontinuities at the heterointerfaces reflect the band offsets between adjacent materials. Near the rear contact, the downward bending of the valence-band edge is accompanied by a localized depletion of holes in WSe_2_. Fig. 6(c) shows that holes carry most of the current in WSe_2_, whereas electrons dominate the current in ZnSe and ITO. Within CIS, the electron and hole current densities vary with position and have opposite signs over part of the absorber. Their sum remains approximately constant at 34.76 mA cm^−2^. The finite hole current near the rear contact shows that the positive local field does not block the total hole current, whose interpretation requires consideration of both drift and diffusion. The concentration profiles in Fig. 6(d) confirm the p-type character of WSe_2_ and CIS and the n-type character of ZnSe and ITO. Away from the interfaces, the hole concentrations approach 10^20^ cm^−3^ in WSe_2_ and 10^19^ cm^−3^ in CIS, consistent with the imposed acceptor densities. Together with the weak field and nearly flat bands, these distributions support a nearly quasi-neutral CIS interior. The combined profiles characterize the local electrostatic and transport conditions at the investigated bias, but do not independently establish reduced hole collection or enhanced recombination at the rear contact.

**Fig. 6:**
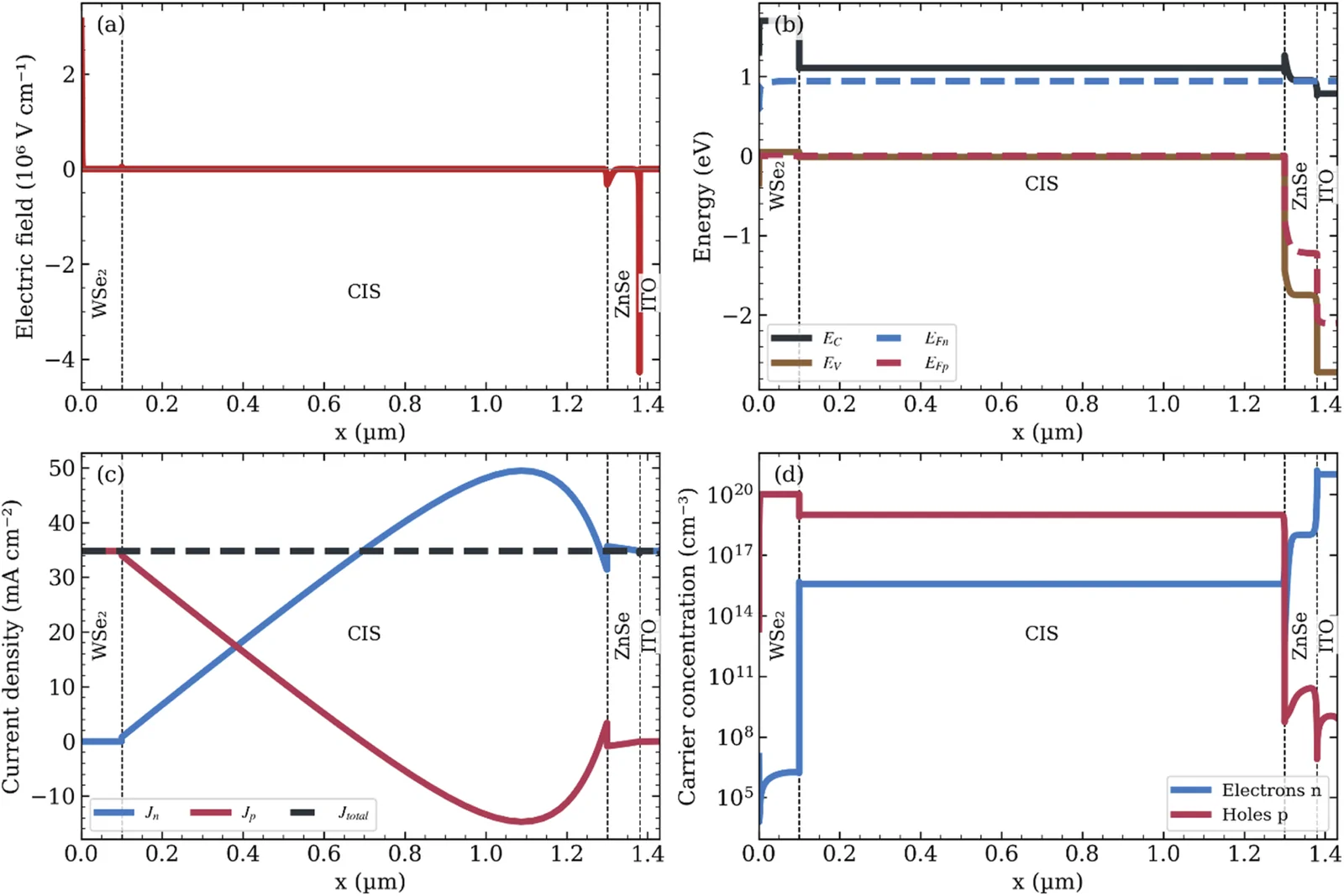
Spatial profiles of (a) electric field, (b) energy bands and quasi-Fermi levels, (c) electron, hole, and total current densities, and (d) carrier concentrations.

### 3.6 Influence of interface defect

To assess the influence of interface defects, four configurations were compared by selectively disabling the defect states at ZnSe/CIS and CIS/WSe_2_. Table 4 shows that disabling the CIS/WSe_2_ defect states produces only minor changes, with a PCE gain of 0.01 percentage points. Disabling the ZnSe/CIS defect states has a more pronounced effect, increasing the PCE by 0.10 percentage points. The highest PCE, 34.42%, is obtained when both interface defect distributions are disabled, corresponding to a total gain of 0.12 percentage points. *V*_oc_, *J*_sc_, and FF also increase slightly. These results indicate greater sensitivity to the ZnSe/CIS defect states under the adopted simulation conditions, although the overall photovoltaic changes remain small. In trap-assisted recombination models, the recombination rate depends on the local electron and hole populations as well as the defect parameters.^30,31^ Energy barriers at heterointerfaces also influence interface recombination and device performance.^32^ These considerations provide possible explanations for the different sensitivities observed here; the photovoltaic parameters alone do not establish their individual contributions.

**Table 4:** Effect of interface defect states on the photovoltaic parameters of the CIS solar cell

Interface defect configuration	*V*_oc_ (V)	*J*_sc_ (mA cm^−2^)	FF (%)	PCE (%)	ΔPCE^a^ (percentage points)
Defect states enabled at both interfaces	0.9240	42.75582	86.82	34.30	—
CIS/WSe_2_ defect states disabled	0.9242	42.75630	86.83	34.31	+0.01
ZnSe/CIS defect states disabled	0.9254	42.78254	86.90	34.40	+0.10
Defect states disabled at both interfaces	0.9257	42.78302	86.91	34.42	+0.12

a Is calculated relative to the configuration with defect states enabled at both interfaces. Only the defect states are disabled; all physical layers and interfaces are retained.

### 3.7 Impedance and modulus spectroscopic analysis

Impedance spectroscopy was used to examine the small-signal AC response of the simulated device. The combined use of complex impedance *Z*(*ω*) and electric modulus *M*(*ω*) provides complementary information and helps distinguish relaxation contributions associated with different electrical processes.

#### 3.7.1 Analysis of the complex impedance at different thickness

Fig. 7(a) shows the negative imaginary part of impedance (−*Z*″) against its real part (*Z*′) for different CIS absorber thicknesses. We observe that as the absorber thickness increases (from 0.2 µm to 1.6 µm), the diameter of the semicircle grows significantly, and the entire semicircle moves horizontally to the right along the *Z*′ axis. The increasing arc diameter indicates a larger resistive contribution. The corresponding capacitance cannot be inferred from the diameter alone and is evaluated through equivalent-circuit fitting.^33^ The shift along the *Z*′ axis also points to a higher overall real impedance for thicker films. Fig. 7(b) shows the negative imaginary part of impedance (−*Z*″) as a function of frequency for different thicknesses. The peak amplitude of −*Z*″ increases significantly with increasing thickness. At the same time, the peak frequency shifts to lower frequencies as thickness increases. For example, the peak is much higher and at a lower frequency for 1.6 µm compared to 0.2 µm. This is consistent with the observation in Fig. 7(a). The larger −*Z*″ peak amplitude is consistent with the increased resistive contribution observed in the Nyquist spectra. The shift toward lower frequencies indicates a longer apparent characteristic relaxation time. Thickness-dependent changes in carrier transport and recombination may contribute to this behavior.

**Fig. 7:**
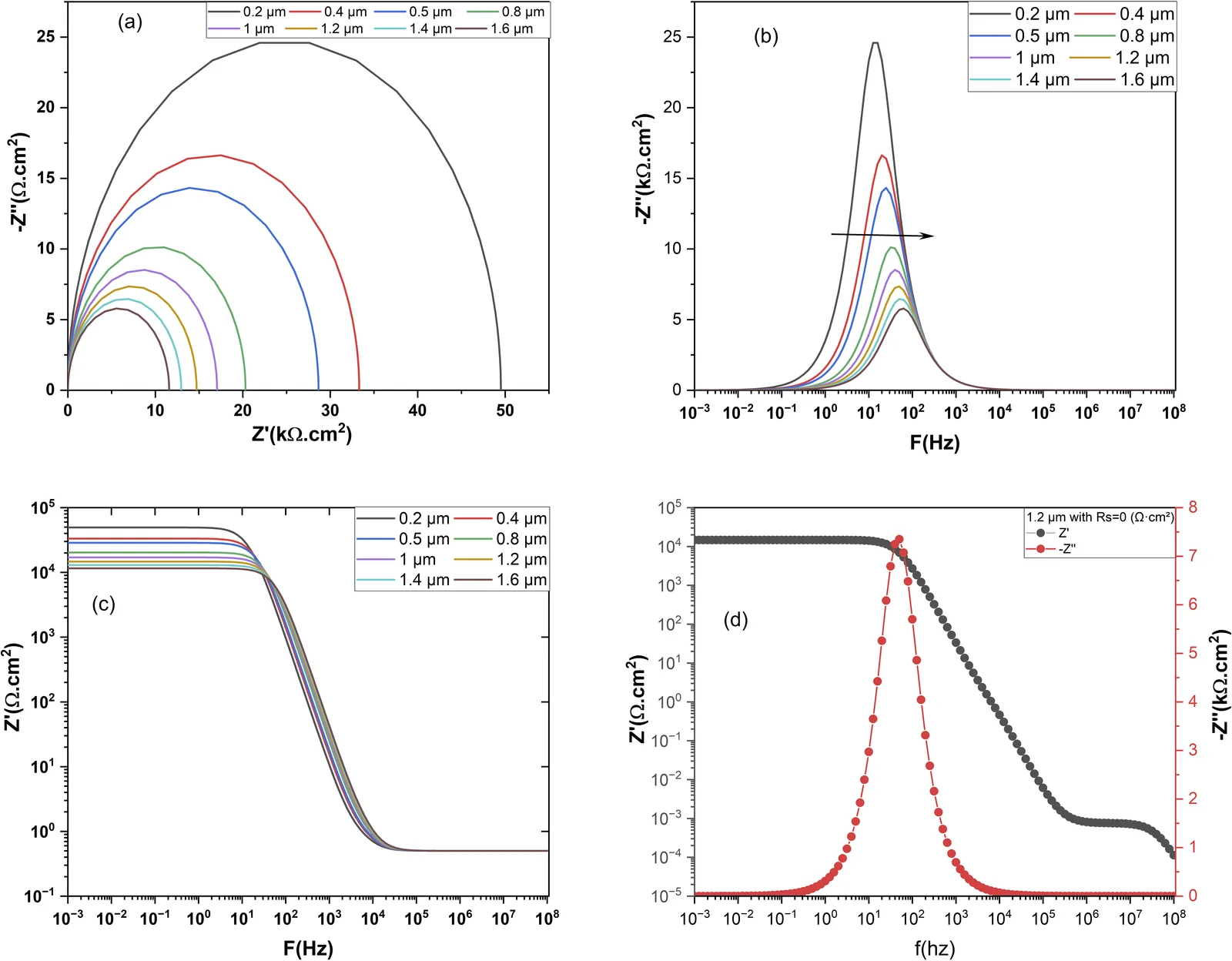
Plot of (a) Nyquist (b and c) Bode at different thickness and Bode for thickness 1.2 µm with *R*_s_ = 0 ohm cm^2^ (d).

Fig. 7(c) shows *Z*′ as a function of frequency for various thicknesses. At low frequencies, below approximately 10^2^ Hz, *Z*′ is high and relatively flat. Its value increases with absorber thickness, indicating a higher low-frequency differential resistance for thicker films. At high frequencies, *Z*′ decreases and the curves approach a common limit, consistent with the same imposed series resistance across the thickness sweep.

Fig. 7(d) shows the real part of impedance (*Z*′) and the negative imaginary part (−*Z*″) as a function of frequency for the optimal absorber thickness of 1.2 µm, with *R*_s_ = 0 Ω cm^2^ to exclude the contribution of the imposed external series resistance. *Z*′ is high at low frequencies and decreases as frequency increases, indicating that slow electrical contributions dominate the low-frequency response. The changes in slope are consistent with overlapping relaxation contributions. In semiconductor solar cells, such responses can involve coupled charge transport, charge accumulation, and recombination.^34^ Meanwhile, the −*Z*″ curve (red line) exhibits a clear peak around 10^2^ Hz, corresponding to the dominant relaxation contribution. This contribution may involve recombination coupled with charge storage, although its assignment specifically to junction recombination requires support from the fitted parameters and their sensitivity to device conditions.

#### 3.7.2 Analysis of the complex modulus at different thickness

To further deepen our analysis and effectively visualize the relaxation processes that might be obscured or masked in traditional impedance plots, we utilized the complex modulus (*M*′ and *M*″) formalism. This complementary representation can help distinguish overlapping electrical contributions from junctions and interfaces, as demonstrated in chalcogenide solar cells^35,36^

Fig. 8(a) and (b) these figures provide an overall view of how the modulus spectra change with absorber thickness. Fig. 8(a), the Modulus Bode plot (*M*″ *vs.* Frequency), shows that as the absorber thickness increases, the amplitude of the dominant *M*″ peak significantly rises, and its frequency shifts towards lower values. Fig. 8(b), shows that the modulus arcs for different absorber thicknesses nearly overlap. The effect of thickness is more apparent in the frequency-dependent spectra in Fig. 8(a).

**Fig. 8:**
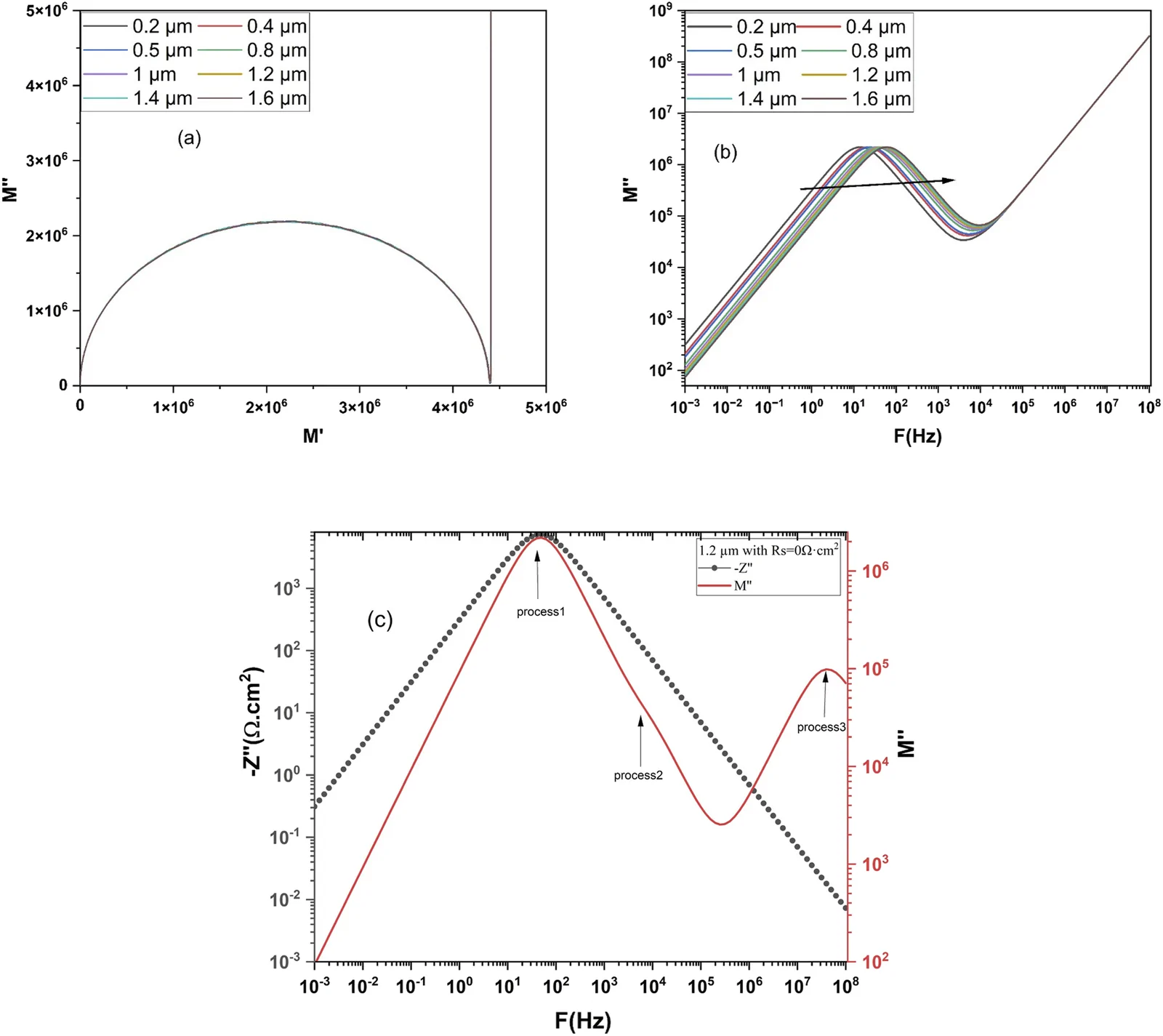
Plot of (a) Nyquist (b) Bode at different thickness and Bode for thickness 1.2 µm with *R*_s_ = 0 ohm cm^2^ (c).

Fig. 8(c) shows −*Z*″ and *M*″ as functions of frequency for the optimal absorber thickness of 1.2 µm, with *R*_s_ = 0 Ω cm^2^. Setting *R*_s_ to zero removes the contribution of the imposed lumped series resistance from this comparison. The −*Z*″ curve exhibits a clear maximum around 10^1^–10^2^ Hz, followed by a decrease at higher frequencies. This peak represents the dominant impedance relaxation, which may include a contribution from junction recombination. The corresponding *M*″ spectrum reveals two resolved maxima separated by an intermediate-frequency shoulder or transition region, as indicated by the arrows:

• Process 1 (low-frequency *M*″ peak): this peak, seen at low frequencies (10^1^–10^2^ Hz), is typically linked to recombination processes, which may originate from deep trap states, bulk and surface recombination, or parasitic shunt channels within the device.

• Process 2 (mid-frequency *M*″ dip/shoulder): this shows a transition area or another relaxation process, often related to charge transport dynamics or different types of trap states.

• Process 3 (high-frequency *M*″ peak): this peak, appearing at higher frequencies (around 10^7^ Hz), means a faster relaxation process, possibly assigned to a fast high-frequency relaxation process associated with the HTL/back-contact interface.

To examine the proposed back-contact contribution, the work function of the rear contact was varied from 4.9 to 5.5 eV while all other simulation parameters were kept unchanged. Fig. 9 shows that the low-frequency maximum remains close to 50 Hz, whereas the high-frequency response changes markedly. Increasing the work function from 4.9 to 5.0 eV shifts the high-frequency maximum from approximately 1 MHz to 39.8 MHz. At 5.1 eV and above, the response continues to rise toward the upper frequency limit, suggesting that the maximum lies beyond the frequency range under investigation. The greater sensitivity of the high-frequency response to this controlled change supports a contribution from the back-contact region to Process 3.

**Fig. 9:**
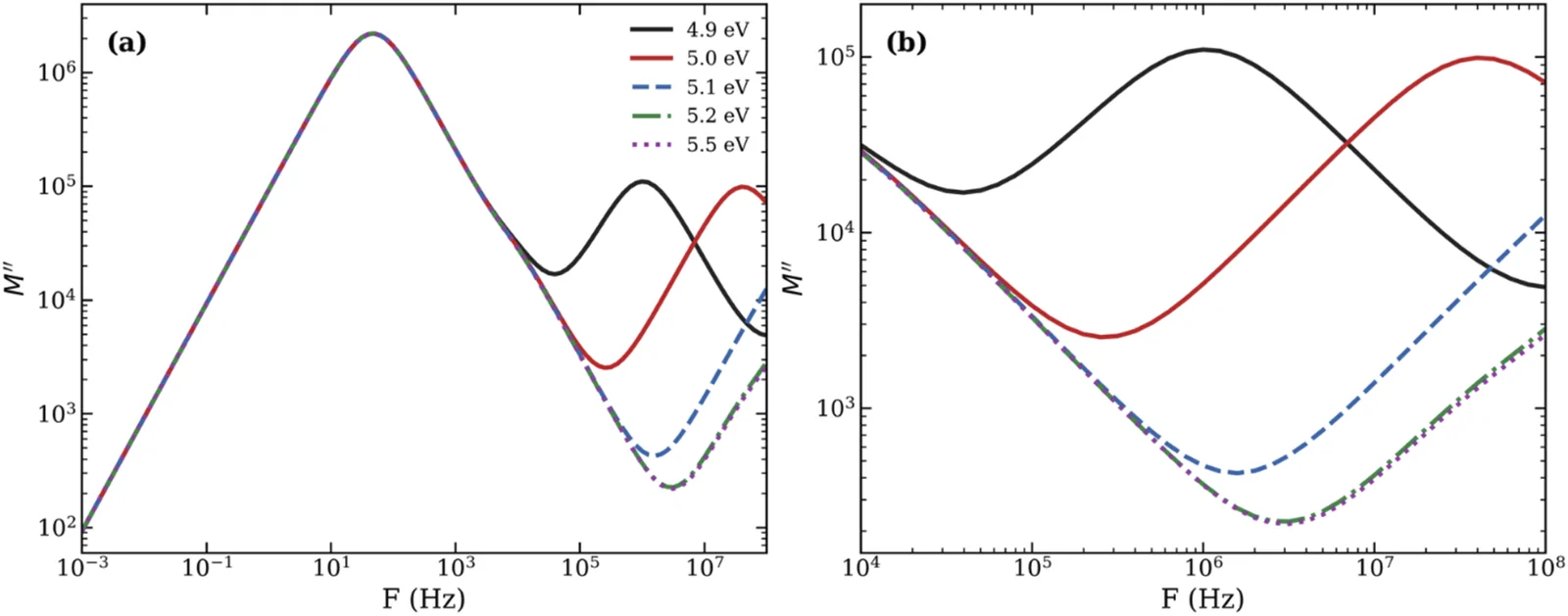
Effect of the back-contact work function on the imaginary electric modulus: (a) full frequency range; (b) enlargement of the high-frequency region.

#### 3.7.3 Analysis of the complex impedance at different series (*R*_s_) and shunt (*R*_sh_) resistances values

Fig. 10(a) displays the imaginary part of the impedance (*Z*″) against the real part (*Z*′). It shows a prominent semicircle that extends along the *Z*′ axis. As the series resistance (*R*_s_) increases (from 0 to 1 Ohm cm^2^), the entire semicircle shifts horizontally to the right along the *Z*′ axis. Crucially, the diameter of this semicircle remains unchanged for all *R*_s_ values. This confirms that the internal processes responsible for the semicircle (such as recombination resistance and junction capacitance) are not directly influenced by changes in series resistance, but *R*_s_ merely adds an ohmic component in series with the overall impedance. The high-frequency intercept with the *Z*′ axis directly corresponds to the value of *R*_s_. Fig. 10(b) illustrates the imaginary part of the impedance (*Z*″) as a function of frequency. All the curves corresponding to different *R*_s_ values perfectly overlap, forming a single, distinct peak. This observation clearly demonstrates that the imaginary component of the impedance is entirely unaffected by changes in series resistance. Since *Z*″ represents the reactive behavior of the solar cell, its invariance confirms that the charge carrier dynamics and energy storage processes contributing to this reactive part are not influenced by the ohmic resistance in series. The peak in *Z*″ indicates a specific frequency where reactive losses are maximized for the dominant RC element in the cell. Fig. 10(c) shows the real part of the impedance (*Z*′) as a function of frequency. At low frequencies (below approximately 10^3^ Hz), all curves for different *R*_s_ values appear to overlap, indicating that the low-frequency resistive behavior is independent of *R*_s_. However, at higher frequencies (above approximately 10^4^ Hz), the *Z*′ curves diverge and flatten out, showing a clear dependence on *R*_s_. The plateau value of *Z*′ directly corresponds to the value of *R*_s_.

**Fig. 10:**
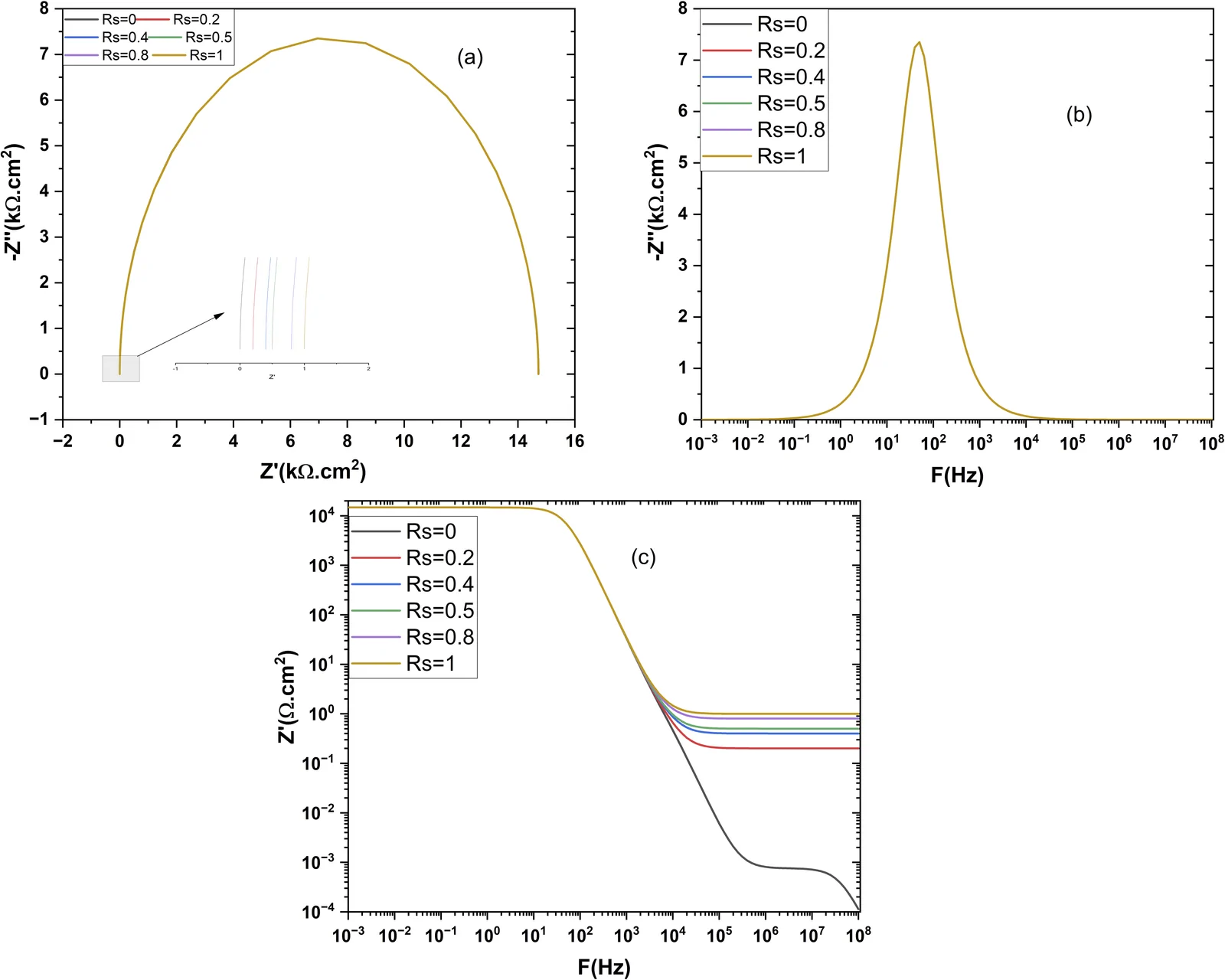
Impedance spectra (a) Nyquist plot (b and c) Bode plots at different series resistances values.

To assess how parallel leakage affects the frequency-dependent electrical response, impedance spectra were calculated for *R*_sh_ values ranging from 10^2^ to 10^11^ Ω cm^2^, with *R*_s_ fixed at 0 Ω cm^2^ and all other simulation parameters kept unchanged. Fig. 11 presents the resulting Nyquist plots and the frequency dependence of the imaginary and real parts of impedance. Fig. 11(a) presents the Nyquist plots (−*Z*″ *versus Z*′) for different shunt resistance values. At low *R*_sh_ values (10^2^ and 10^3^ Ω cm^2^), the semicircle is small and lies close to the origin. The parallel leakage path reduces the effective resistance measured across the device. As *R*_sh_ increases, the semicircle diameter grows before approaching a limiting value, indicating that the influence of shunt leakage becomes progressively less significant. Fig. 11(b) shows the corresponding −*Z*″ spectra. At low *R*_sh_, the peak amplitude is small and the maximum occurs at higher frequencies. Increasing *R*_sh_ raises the peak amplitude and shifts it toward lower frequencies. The curves for 10^9^ and 10^11^ Ω cm^2^ nearly overlap, indicating that further increases in *R*_sh_ have little effect on the dominant response. This behavior is consistent with an increase in the effective resistance and the associated RC time constant as parallel leakage decreases. The frequency shift does not, by itself, establish changes in trapping/detrapping kinetics or carrier recombination. Fig. 11(c) shows the frequency dependence of *Z*′. In the low-frequency region, *Z*′ increases with *R*_sh_ before approaching a limiting value, consistent with the decreasing contribution of parallel leakage to the differential resistance. At sufficiently high frequencies, the curves nearly overlap, indicating that the influence of *R*_sh_ becomes negligible in this region.

**Fig. 11:**
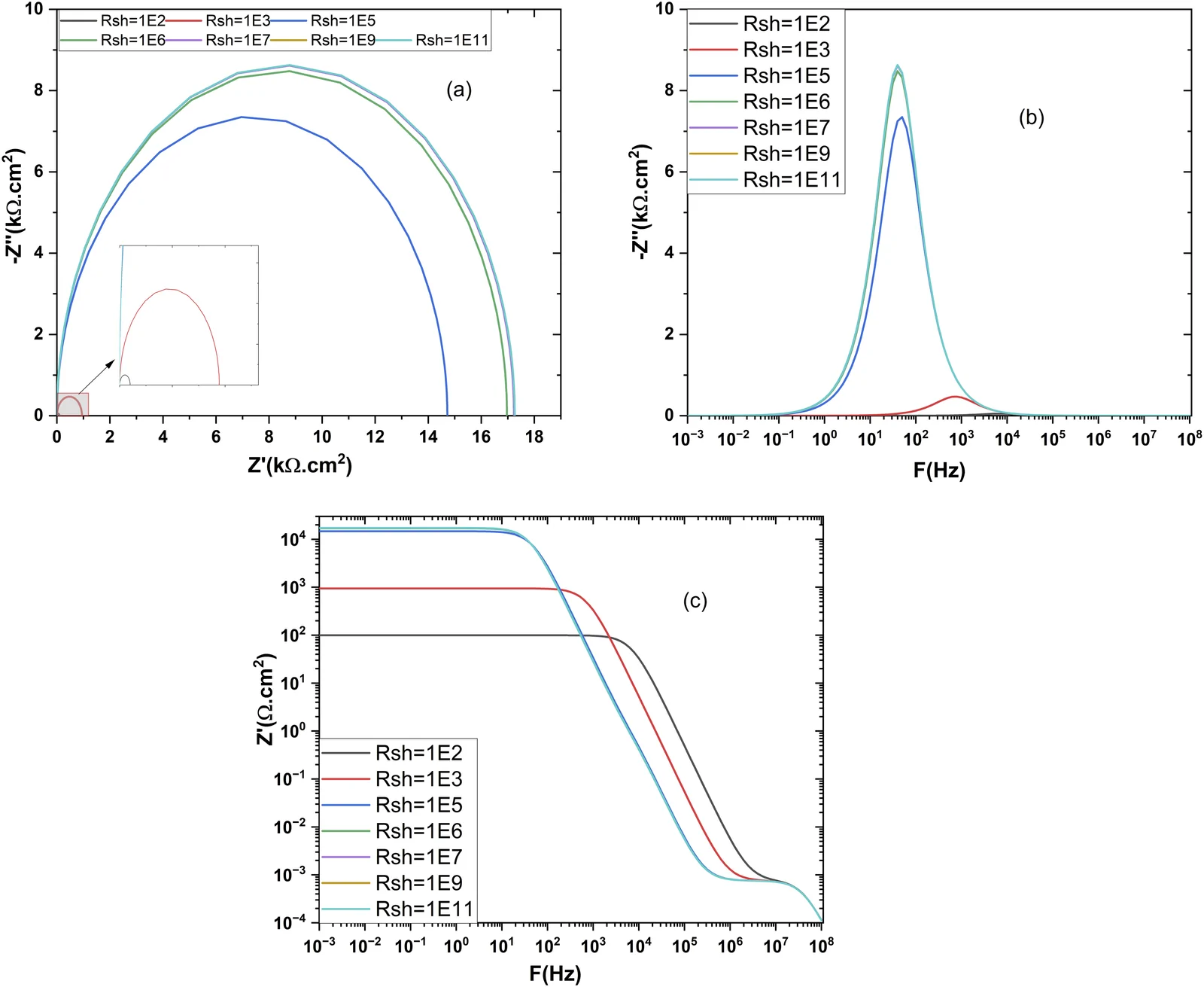
Impedance spectra (a) Nyquist plot (b and c) Bode plots at different Shunt resistances values.

#### 3.7.4 Analysis of the modulus at different series and shunt resistances values

In Fig. 12(a), using the modulus function in the Nyquist diagram helps us see how series resistance (*R*_s_) affects processes at medium and high frequencies. The size of the semicircle for the low-frequency process does not change. When *R*_s_ is 0 Ω cm^2^, the low- and high-frequency features and the intermediate-frequency shoulder are more clearly resolved. However, starting from higher *R*_s_ values (especially from *R*_s_ = 0.2 Ohm cm^2^), the medium and high-frequency processes (Process 2 and 3) begin to fade and become harder to see or are hidden. Fig. 12(b) in the Bode diagrams further supports and shows this behavior. When *R*_s_ is 0 Ohm cm^2^, the two maxima and the intermediate-frequency shoulder are more clearly visible. But as *R*_s_ gets higher, the peaks for Process 2 and 3 are hidden or covered by the strong response from *R*_s_ itself. This analysis shows that series resistance (*R*_s_) has a strong effect on how well we can see and determine the recombination process (Process 1), the charge transport process (Process 2), and the relaxation at the interface (Process 3), especially at medium and higher frequencies. This masking arises because the series-resistance contribution to *M*″ increases with frequency, making the weaker features harder to distinguish without implying suppression of the underlying processes.

**Fig. 12:**
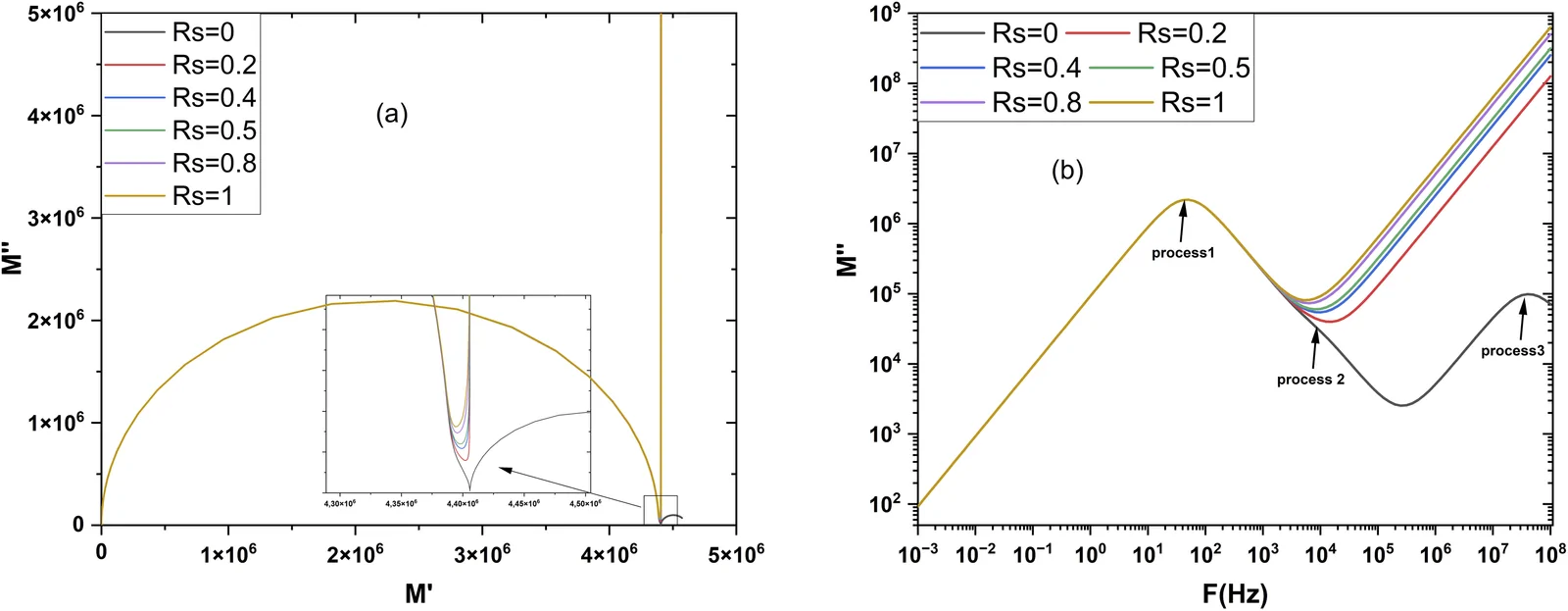
Modulus spectra (a) Nyquist plot and (b) Bode plots of solar cell at different serie resistances values.

Fig. 13(a and b) show the modulus response of the solar cell for different values of shunt resistance (*R*_sh_). As seen in Fig. 13(a), the Nyquist plot highlights that varying *R*_sh_ changes the diameter of the semicircles at low and medium frequencies. On the other hand, the high-frequency semicircle remains nearly unchanged, suggesting that it is weakly affected by *R*_sh_ over the investigated range. Fig. 13(b) shows the imaginary part of the modulus (*M*″) against frequency. For very low *R*_sh_ values (10^2^ and 10^3^ Ω cm^2^), the low- and intermediate-frequency features are less clearly resolved. This behavior is consistent with the low-resistance parallel path provided by *R*_sh_, which modifies the overall electrical response and makes overlapping relaxation contributions harder to distinguish. As *R*_sh_ increases, starting from 10^5^ Ω cm^2^, a clearer peak appears, its height increases, and its frequency shifts toward lower values. This evolution indicates a change in the effective electrical relaxation as parallel leakage decreases. A contribution from carrier transport may be present, but the peak cannot be assigned uniquely to transport from the modulus spectra alone. The corresponding resistive and capacitive contributions are examined through equivalent-circuit analysis in the following section.

**Fig. 13:**
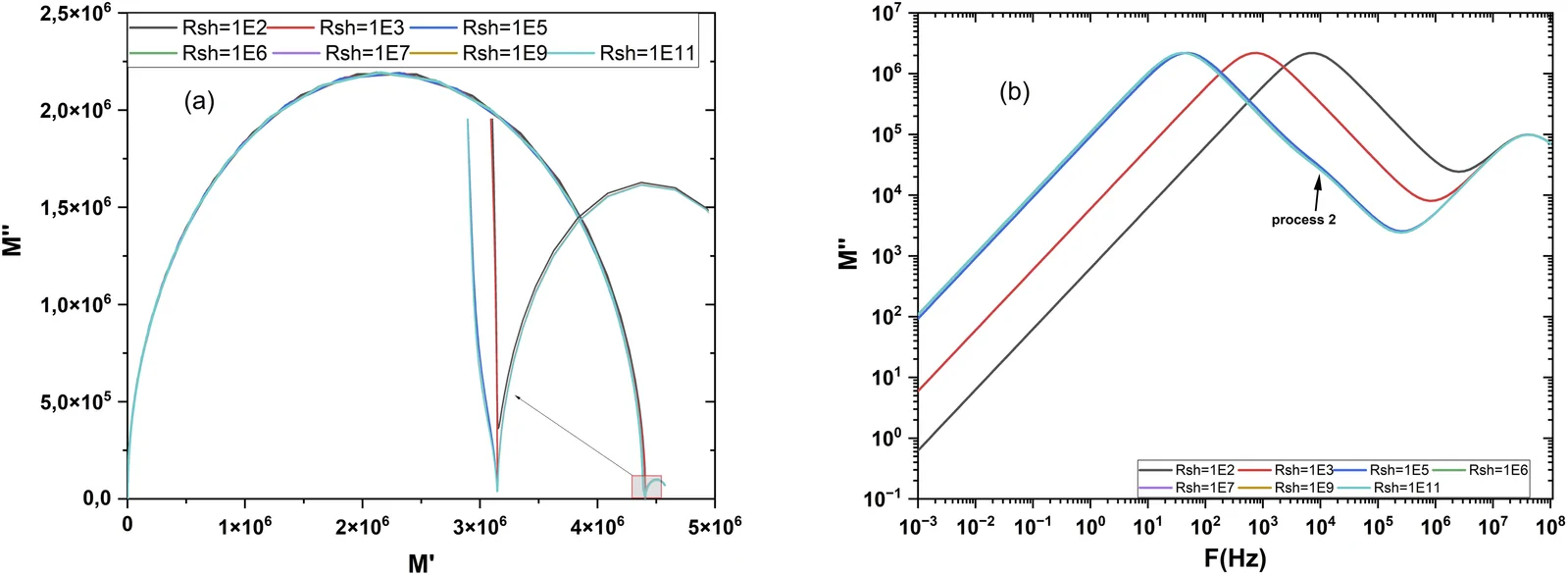
Modulus spectra (a) Nyquist plot and (b) Bode plots of solar cell at different shunt resistances values.

#### 3.7.5 Investigation of interface defects *via* impedance spectroscopy

Fig. 14–16 provide further insight into the effect of interface defects on the impedance response of the CIS solar cell. In Fig. 14, the comparison between the device with and without both absorber-related interfaces shows that the main −*Z*″ peak remains nearly in the same frequency range, indicating that the dominant relaxation process is globally preserved. However, slight changes in the amplitude of −*Z*″ and in the slope of *Z*′ can still be observed, which suggests that the interfaces modify the contribution of the dominant process without introducing a completely new relaxation mechanism. The differences remain limited, showing that the absorber-related interfaces do not strongly control the main low-frequency response. In Fig. 15, removing the HTL/absorber interface produces only very small changes in *Z*′, −*Z*″, and *M*″, indicating that this interface has only a minor influence on the overall AC behavior of the device. This means that the recombination-related and transport-related processes remain almost unchanged when this interface is removed. In Fig. 16, the removal of the ETL/absorber interface leads to a slightly more noticeable variation in the spectra compared with Fig. 15, especially in the intermediate-frequency region, which suggests that this interface contributes somewhat more to the electrical response of the device. Nevertheless, the high-frequency contribution remains nearly unchanged in all three figures, indicating that the fast high-frequency response is not significantly governed by the HTL/absorber or ETL/absorber interfaces. In contrast, the observed variations in *Z*′ and −*Z*″ show that these absorber-related interfaces do contribute to the recombination- and transport-related responses of the device. Overall, Fig. 14–16 suggest that the studied interfaces mainly influence the low- and intermediate-frequency contributions, with the ETL/absorber interface showing a slightly more noticeable effect than the HTL/absorber one, while the high-frequency response remains essentially preserved.

**Fig. 14:**
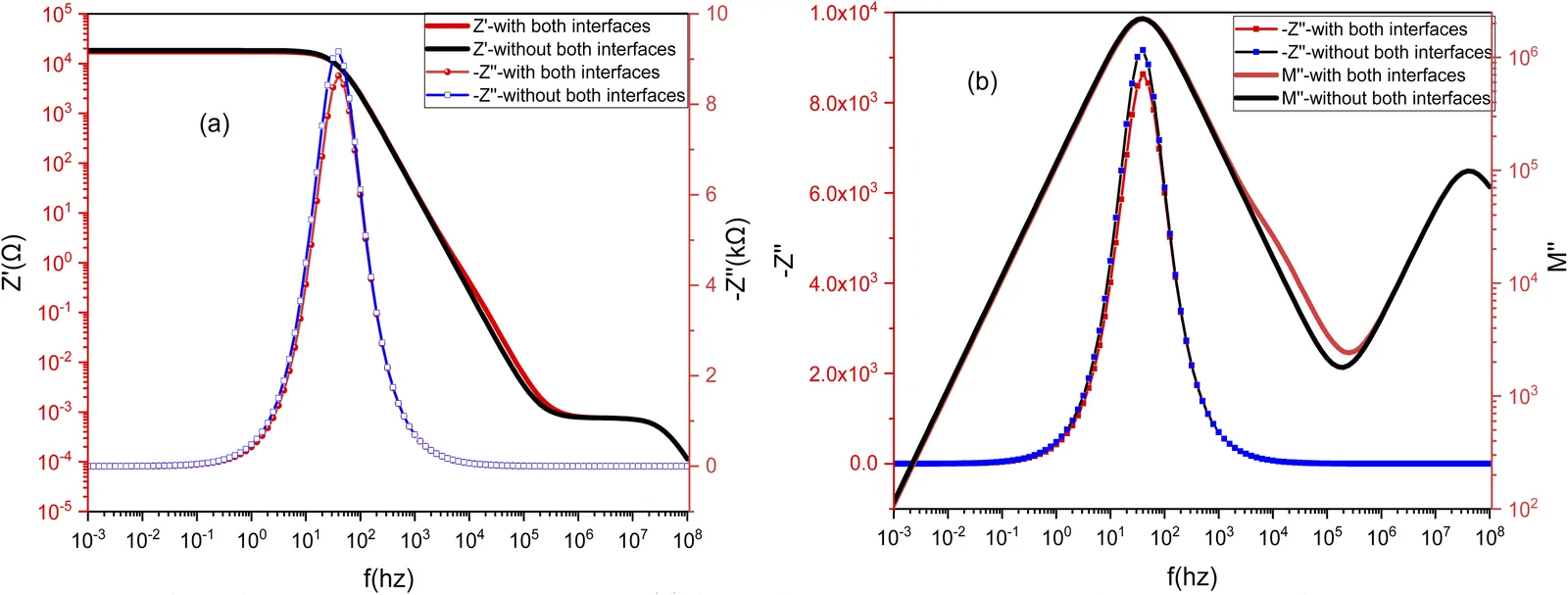
Interface defect analysis by impedance spectroscopy: (a) *Z*′ and −*Z*″ spectra with and without interfaces; (b) −*Z*″ and *M*″ spectra with and without interfaces.

**Fig. 15:**
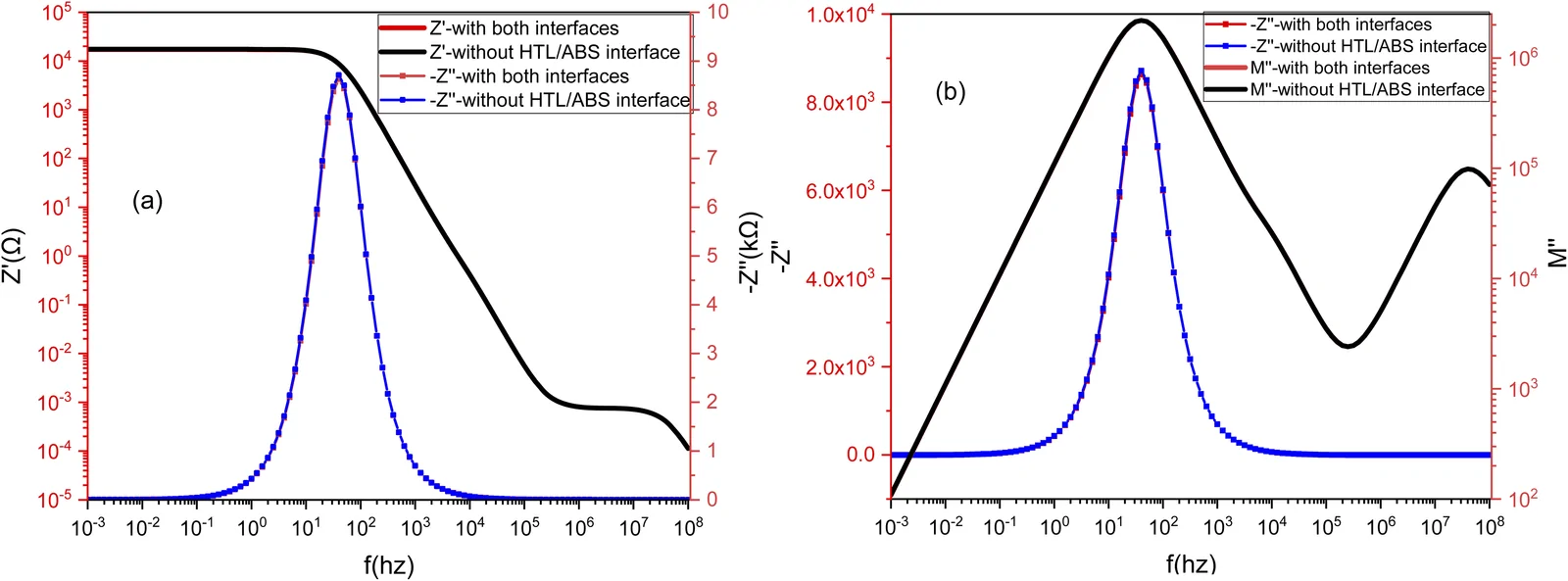
Effect of the HTL/absorber interface on the impedance response: (a) *Z*′ and −*Z*″ spectra with and without the HTL/absorber interface; (b) −*Z*″ and *M*″ spectra with and without the HTL/absorber interface.

**Fig. 16:**
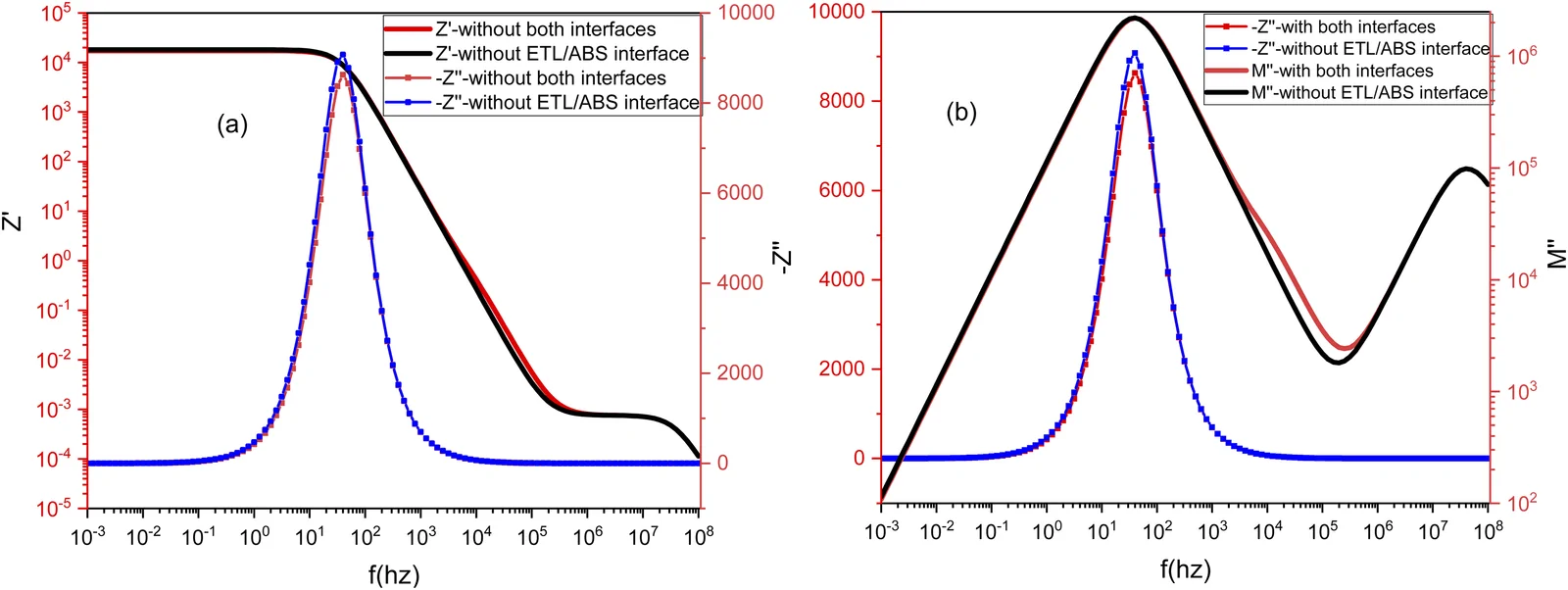
Effect of the ETL/absorber interface on the impedance response: (a) *Z*′ and −*Z*″ spectra with and without the ETL/absorber interface; (b) −*Z*″ and *M*″ spectra with and without the ETL/absorber interface.

### 3.8 Electrical equivalent circuit modeling of CIS solar cells

Chalcopyrite (CIS) solar cells can also be described using an electrical equivalent circuit. In this approach, the complex physical mechanisms are expressed through basic elements such as resistors and capacitors.

• Resistors (R1, R2, R3): represent the losses caused by charge transport resistance and recombination in different regions of the device.

• Capacitors (C1, C2, C3): correspond to charge storage, interfacial polarization, and dielectric properties.

This method makes it possible to extract important parameters, including the series resistance, shunt resistance, and capacitance contributions. Studying these values is useful to understand efficiency losses and to optimize the device design. In summary, equivalent circuit analysis provides a direct link between experimental impedance spectroscopy measurements and the internal charge transport and recombination processes in CIS solar cells. This connection is valuable for improving performance and guiding further development.

The circuit (Fig. 17) is modeled as a chain of three RC branches connected in series. Each branch has a resistor *R*_*i*_ in parallel with a capacitor *C*_*i*_ (*i* = 1, 2, 3).

**Fig. 17:**
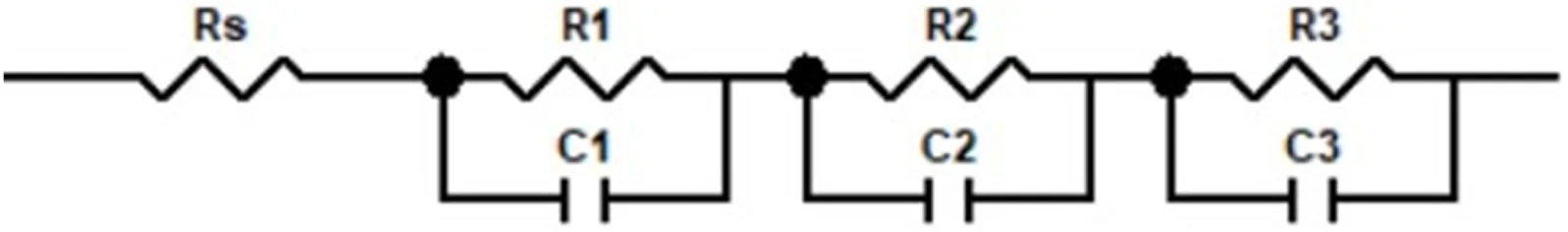
Electrical equivalent circuit.

The impedance of one RC branch is:1
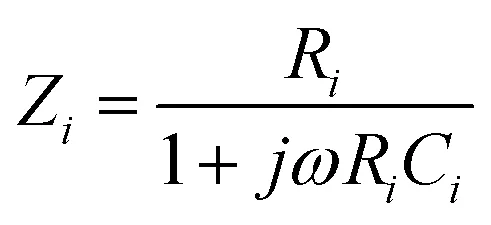


Using the relaxation time:

2 *τ*_*i*_ = *R*_*i*_*C*_*i*_

Then it becomes:3
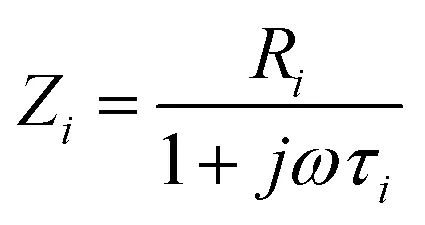
Since the three branches are in series, the total impedance is:4
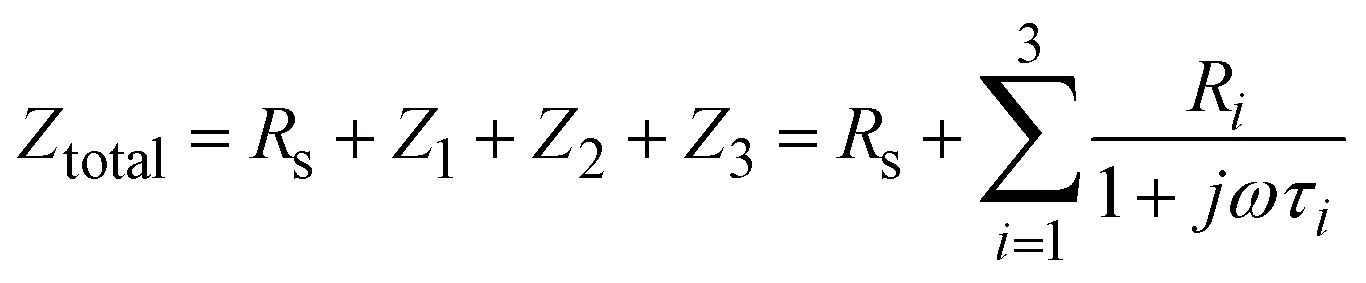


Each RC branch impedance can be expressed in algebraic form (real and imaginary parts) by multiplying numerator and denominator by the complex conjugate:5
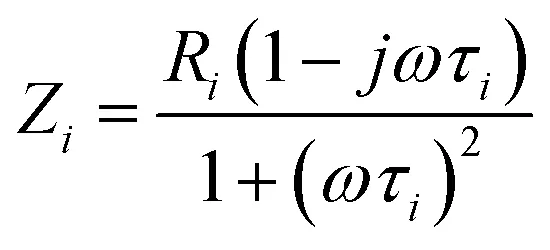


From this, the components are:6
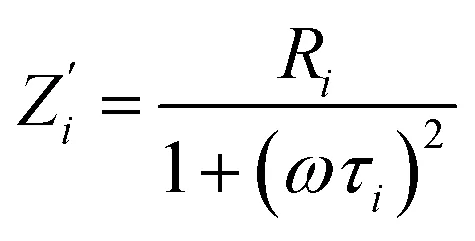
7
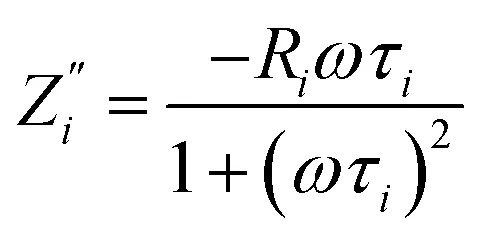
Thus, the total real and imaginary parts of the impedance are:8
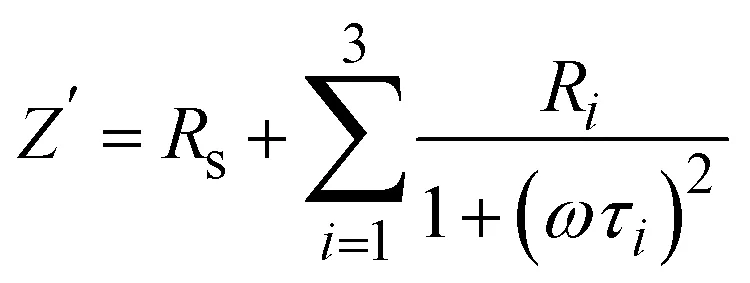
9
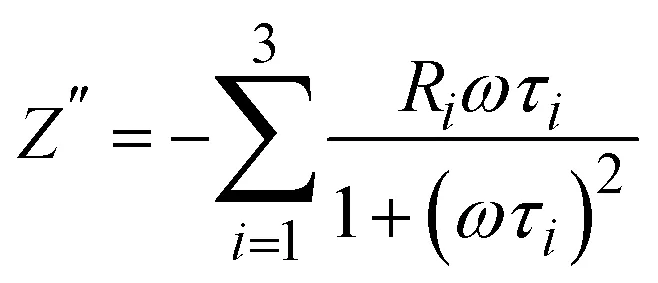


#### 3.8.1 Relationship between impedance and modulus

The complex electric modulus M*(*ω*) is usually introduced as the reciprocal of the complex permittivity. In practice, it can also be formulated directly in terms of the impedance:

10 M*(*ω*) = *jωC*_0_Z*(*ω*)

where Z*(*ω*) = *Z*′ + *jZ*″ is the complex impedance, 
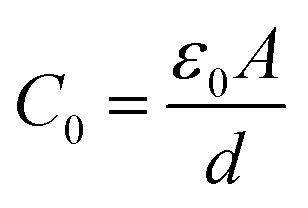
 is the geometrical capacitance of the device (cell area *A*, thickness *d*), and *ω* = 2π*f* is the angular frequency.

11 *M*′(*ω*) = −*ωC*_0_*Z*″(*ω*), *M*″(*ω*) = *ωC*_0_*Z*′(*ω*)

This shows a simple correspondence: the real part of the modulus is proportional to the imaginary part of the impedance, while the imaginary part of the modulus follows the real part of the impedance.12
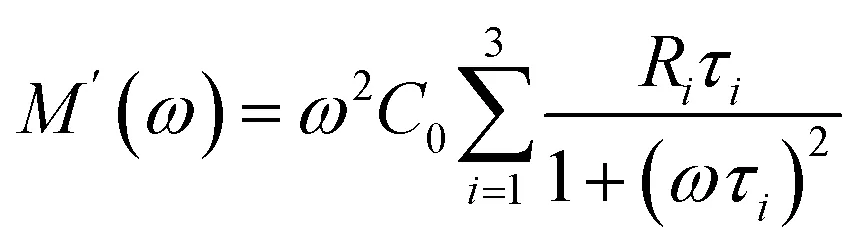
13
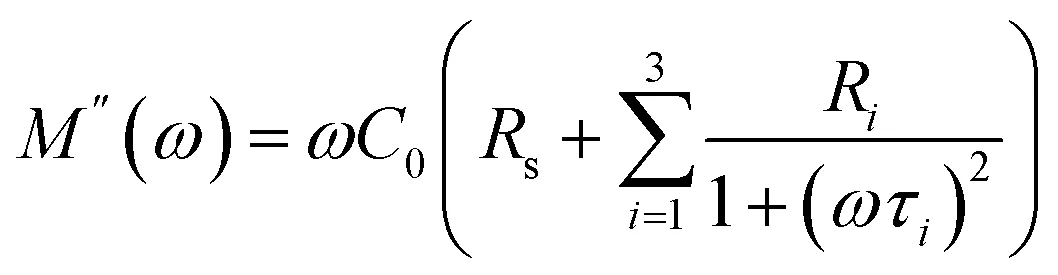


These expressions provide direct access to relaxation dynamics and the role of series resistance, making the modulus representation a complementary tool to the impedance formalism.

#### 3.8.2 Comparison of equivalent-circuit models

To assess whether three RC branches are needed to describe the simulated response, equivalent circuits containing one, two, and three parallel RC branches connected in series were compared. The comparison considers agreement across the full frequency range and in both impedance and modulus representations, since reproducing the dominant impedance peak alone may conceal deviations associated with weaker contributions. Fig. 18 presents this comparison for a CIS absorber thickness of 1.2 µm over the frequency range under investigation. Fig. 18(a) shows that the one-branch model reproduces the dominant low-frequency response but deviates substantially from *Z*′ at higher frequencies. Adding a second branch improves the agreement, although deviations remain in the intermediate-frequency region. These differences are less apparent in −*Z*″, shown in Fig. 18(b), where the dominant peak is closely reproduced by all three models. The modulus representation in Fig. 18(c) makes the weaker contributions easier to distinguish: the one-branch model does not reproduce the high-frequency maximum, whereas the two-branch model captures this feature but retains deviations around the intermediate-frequency transition. The three-branch model provides the closest agreement across these representations. Fig. 18(d) presents the frequency-dependent relative complex error between the fitted and simulated impedance. The complex residuals were normalized by the magnitude of the simulated impedance, and their RMS values were calculated over all frequency points and expressed as percentages. The resulting errors are 0.8946%, 0.1363%, and 0.00452% for the one-, two-, and three-branch models, respectively. The lower residuals and improved reproduction of the intermediate- and high-frequency features support retaining the three-branch circuit among the models examined. Impedance and modulus are transformations of the same electrical response, but their complementary representations reveal deviations that are difficult to distinguish from the dominant impedance peak alone. The physical interpretation of the fitted branches is subsequently examined through their sensitivity to controlled changes in device parameters.

**Fig. 18:**
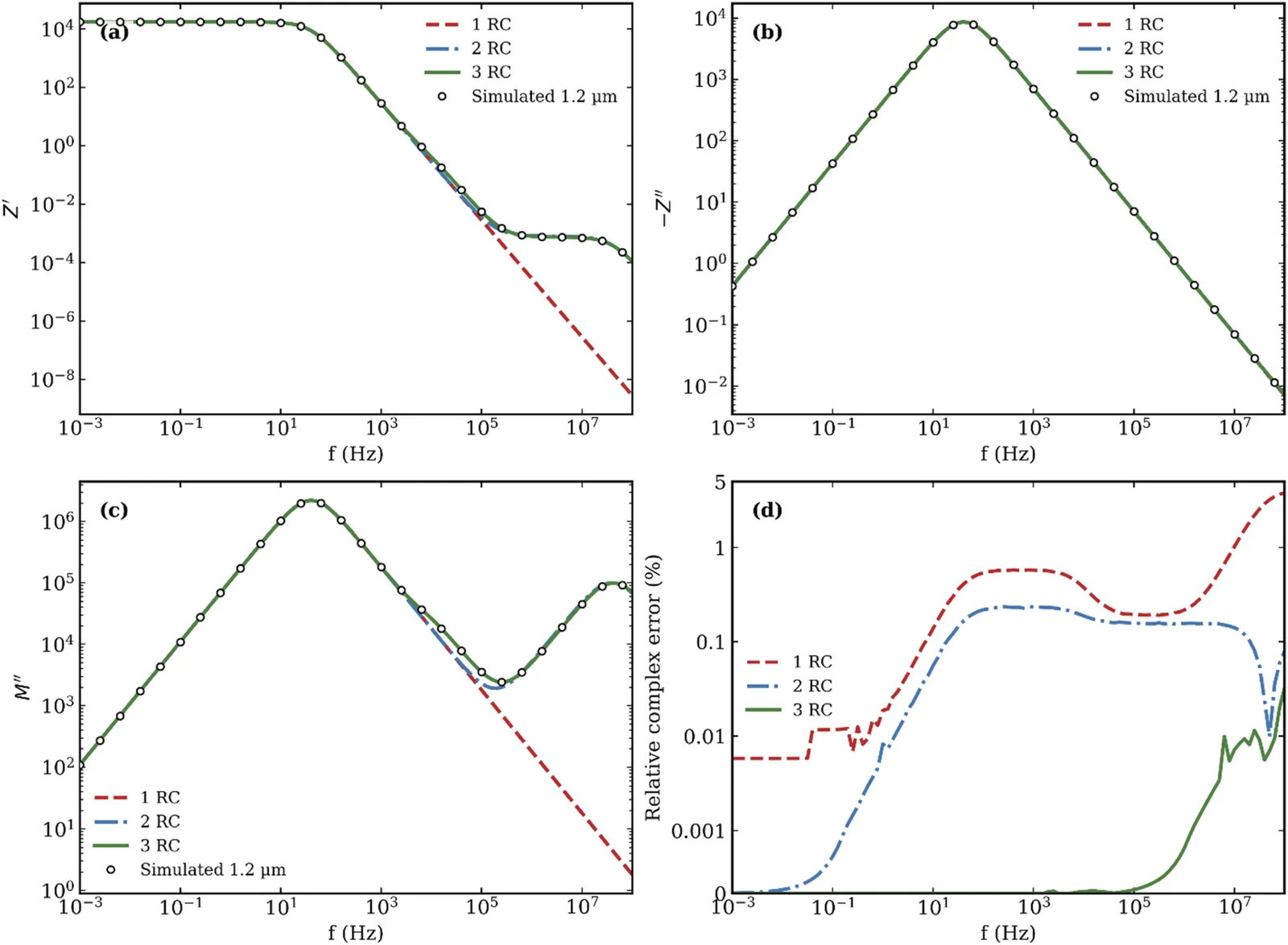
Comparison of one-, two-, and three-branch RC fits with the simulated response at a CIS thickness of 1.2 µm: (a) *Z*′, (b) −*Z*″, (c) *M*″, and (d) relative complex error.

#### 3.8.3 Impedance process deconvolution

The impedance process deconvolution is presented in Fig. 19. In Fig. 19(a), the excellent agreement between the simulated data and the fitted response confirms the validity of the equivalent circuit model in accurately reproducing the device behavior over the entire frequency range. The spectrum is characterized by a dominant peak centered in the intermediate frequency region, indicating that the overall response is governed by a main relaxation process. To further elucidate the origin of this behavior, the total impedance was decomposed into individual contributions, as shown in Fig. 19(b). The results reveal that the response is strongly dominated by Process 1 (R1C1), which exhibits the highest amplitude and defines the global shape of the spectrum. In contrast, Processes 2 (R2C2) and 3 (R3C3) present significantly lower magnitudes and are only observable on a logarithmic scale, reflecting their weaker contribution to the overall impedance. These processes are associated with faster mechanisms occurring at higher frequencies, likely related to charge transport and interfacial phenomena, whereas the dominant Process 1 is attributed to the main recombination dynamics within the device. Overall, this deconvolution highlights the coexistence of multiple relaxation mechanisms with distinct time constants, while emphasizing that the device performance is primarily controlled by the dominant intermediate-frequency process.

**Fig. 19:**
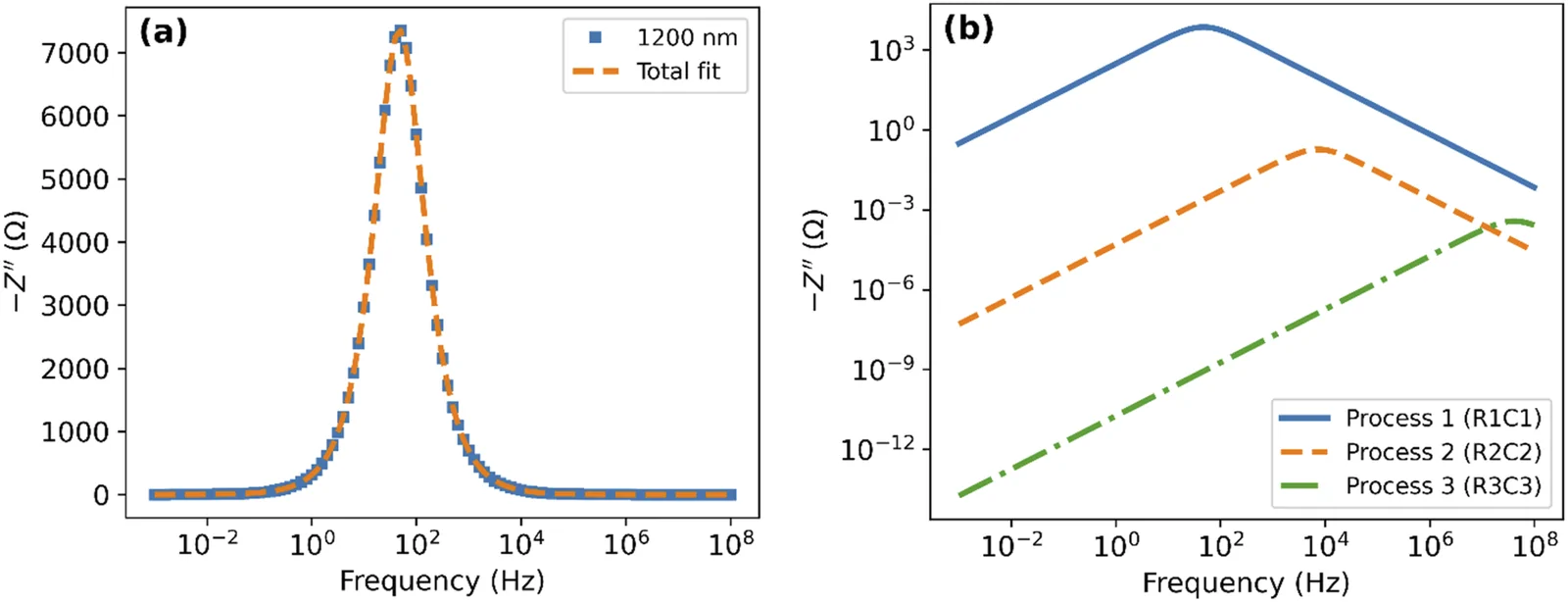
Impedance process deconvolution: (a) comparison between experimental data and fitted response, and (b) individual contributions of the different relaxation processes (R1C1, R2C2, and R3C3).

#### 3.8.4 Correlation between the time constants of charge carriers and the efficiency (PCE)

To examine the relationship between the fitted relaxation times and photovoltaic performance, Fig. 20 and 21 present the evolution of *R*_*i*_, *C*_*i*_, *τ*_*i*_, and PCE with CIS absorber thickness. Examining the resistances and capacitances separately helps explain the changes in each time constant, *τ*_*i*_ = *R*_*i*_*C*_*i*_. The relative changes reported below are calculated with respect to the values at 0.2 µm. As the CIS thickness increases from 0.2 to 1.6 µm, *R*_1_ decreases from approximately 49.5 to 11.6 kΩ cm^2^ (Δ*R*_1_ = −76.6%), while *C*_1_ remains close to 228 nF cm^−2^ (Δ*C*_1_ = +0.13%). Consequently, *τ*_1_ decreases from approximately 11.27 to 2.64 ms, mainly because of the reduction in *R*_1_. This trend is consistent with a recombination-related contribution: a thicker absorber provides a larger volume in which bulk recombination can occur, potentially increasing the differential recombination current and lowering the corresponding resistance. The nearly constant *C*_1_ indicates little variation in the effective charge-storage response represented by this branch. Over the same thickness range, *R*_2_ increases from approximately 0.295 to 0.377 Ω cm^2^ (Δ*R*_2_ = +28.0%), whereas *C*_2_ decreases from 73.6 to 57.0 µF cm^−2^ (Δ*C*_2_ = −22.5%). The increasing resistance is compatible with a transport-related contribution, as increasing absorber thickness extends the distance over which carriers must be collected. These opposing variations largely compensate, leaving *τ*_2_ close to 21.6 µs, with a relative decrease of only 0.83%. Its near stability therefore reflects compensating changes in the fitted resistance and capacitance. In contrast, *R*_3_ and *C*_3_ vary by less than 1% (Δ*R*_3_ = +0.66% and Δ*C*_3_ = −0.59%), keeping *τ*_3_ close to 3.86 ns. This weak dependence on CIS thickness, together with the sensitivity of the high-frequency response to the rear-contact work function, supports the proposed association of this branch with the back-contact region.The comparison with PCE shows that efficiency initially increases despite the decrease in *R*_1_ and *τ*_1_. This is consistent with the improved long-wavelength QE and increasing *J*_sc_ discussed previously: the photocurrent gain initially outweighs the accompanying decreases in *V*_oc_ and FF. At thicknesses above 1.2 µm, further photocurrent gains become insufficient to compensate for these decreases, resulting in a slight reduction in PCE. Thus, 1.2 µm gives the highest simulated efficiency among the investigated thicknesses. The fitted circuit parameters provide complementary insight into this balance, while their characteristic times describe the electrical relaxation response at the AC operating point.

**Fig. 20:**
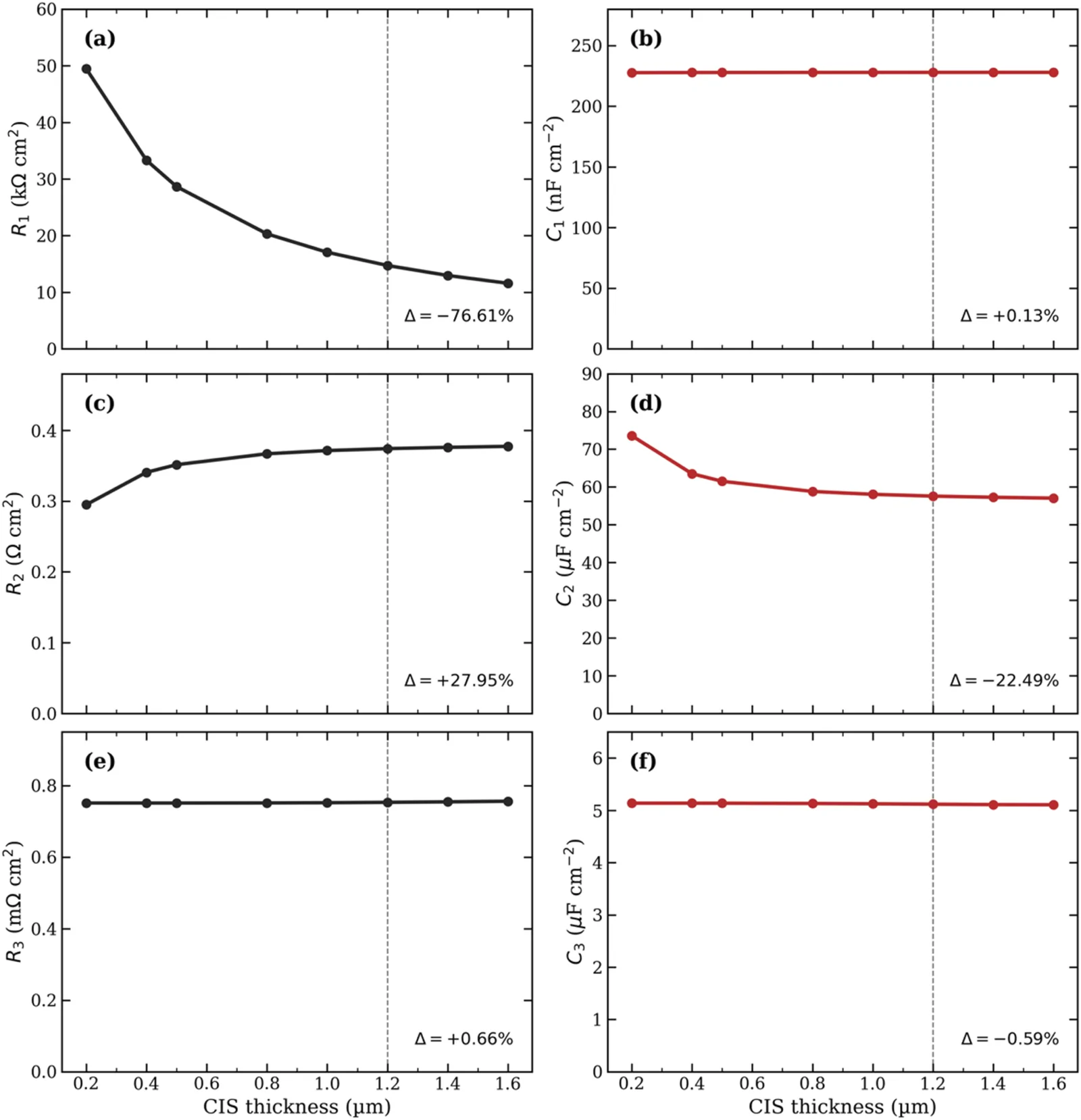
Effect of CIS absorber thickness on the fitted equivalent-circuit parameters: (a) R1, (b) C1, (c) R2, (d) C2, (e) R3, and (f) C3.

**Fig. 21:**
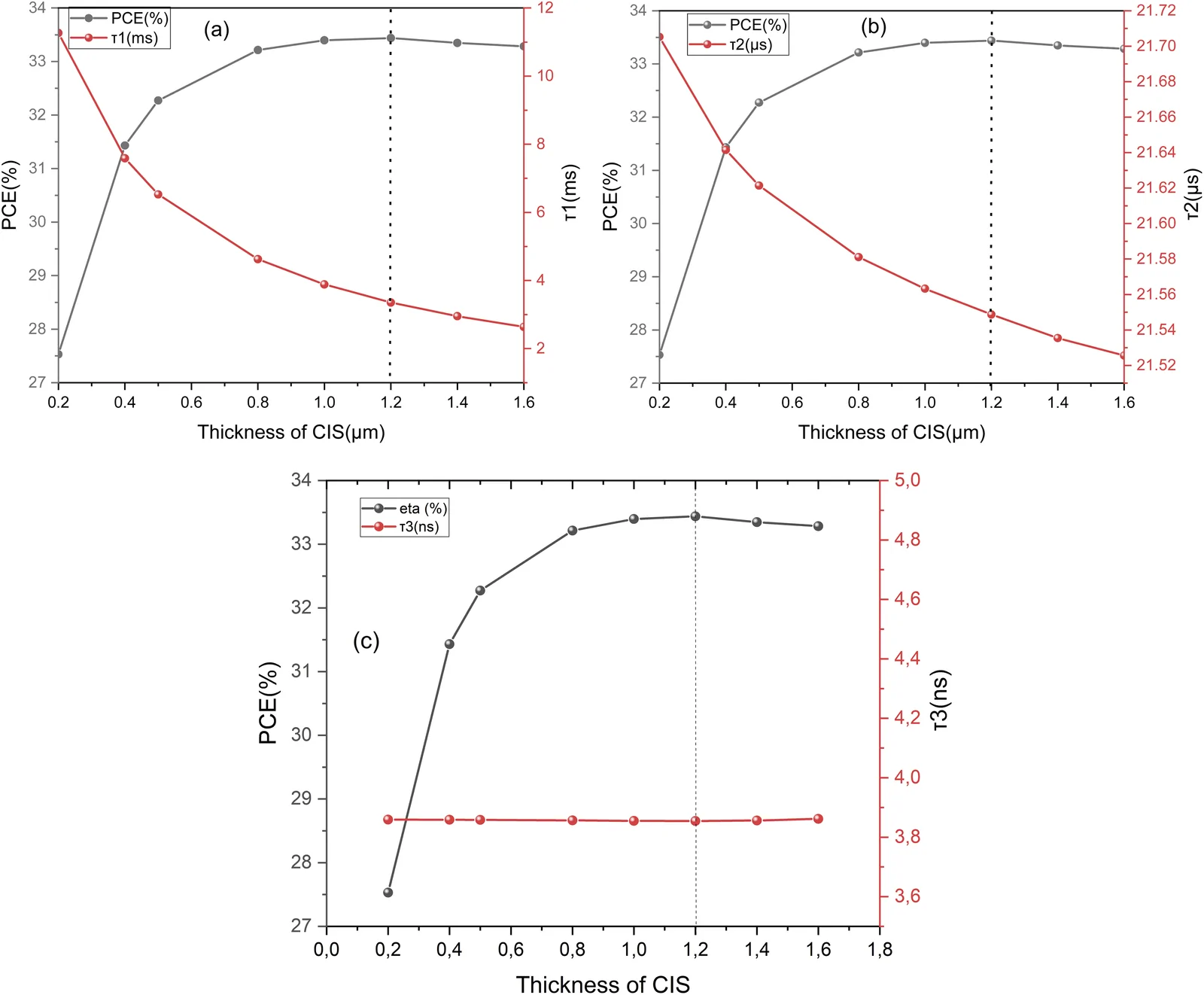
Effect of CIS absorber thickness on photovoltaic efficiency (PCE) and relaxation times: (a) *τ*_1_, (b) *τ*_2_, and (c) *τ*_3_.

Fig. 22(a) illustrates the variation of PCE together with the recombination-related time constant *τ*_1_ as a function of shunt resistance (*R*_sh_). At low *R*_sh_, *τ*_1_ is short, consistent with the reduced effective resistance associated with parallel leakage. As *R*_sh_ increases from 10^2^ to 10^11^ Ω cm^2^, *τ*_1_ rises from approximately 0.022 to 3.93 ms. PCE approaches a plateau around 10^5^ Ω cm^2^, whereas *τ*_1_ stabilizes more gradually at higher *R*_sh_ values. These results indicate that the effective time constant of the recombination-related branch is also influenced by parallel leakage. Fig. 22(b) shows the variation of the transport-related time constant *τ*_2_ and the corresponding PCE. As *R*_sh_ increases from 10^2^ to 10^3^ Ω cm^2^, *τ*_2_ decreases from approximately 26.45 to 21.54 µs and then remains nearly constant. This stability indicates that the intermediate-frequency branch becomes weakly sensitive to further increases in *R*_sh_. PCE nevertheless continues to improve up to approximately 10^5^ Ω cm^2^, reflecting the progressive reduction of leakage losses affecting *V*_oc_ and FF. Fig. 22(c) shows that *τ*_3_ remains close to 3.85 ns throughout the investigated *R*_sh_ range. This weak sensitivity to parallel leakage, together with the sensitivity of the high-frequency response to the rear-contact work function, supports the proposed association of this branch with the back-contact region. Overall, increasing *R*_sh_ improves photovoltaic performance primarily by reducing leakage losses, while its strongest influence on the fitted relaxation times occurs in the low-frequency branch.

**Fig. 22:**
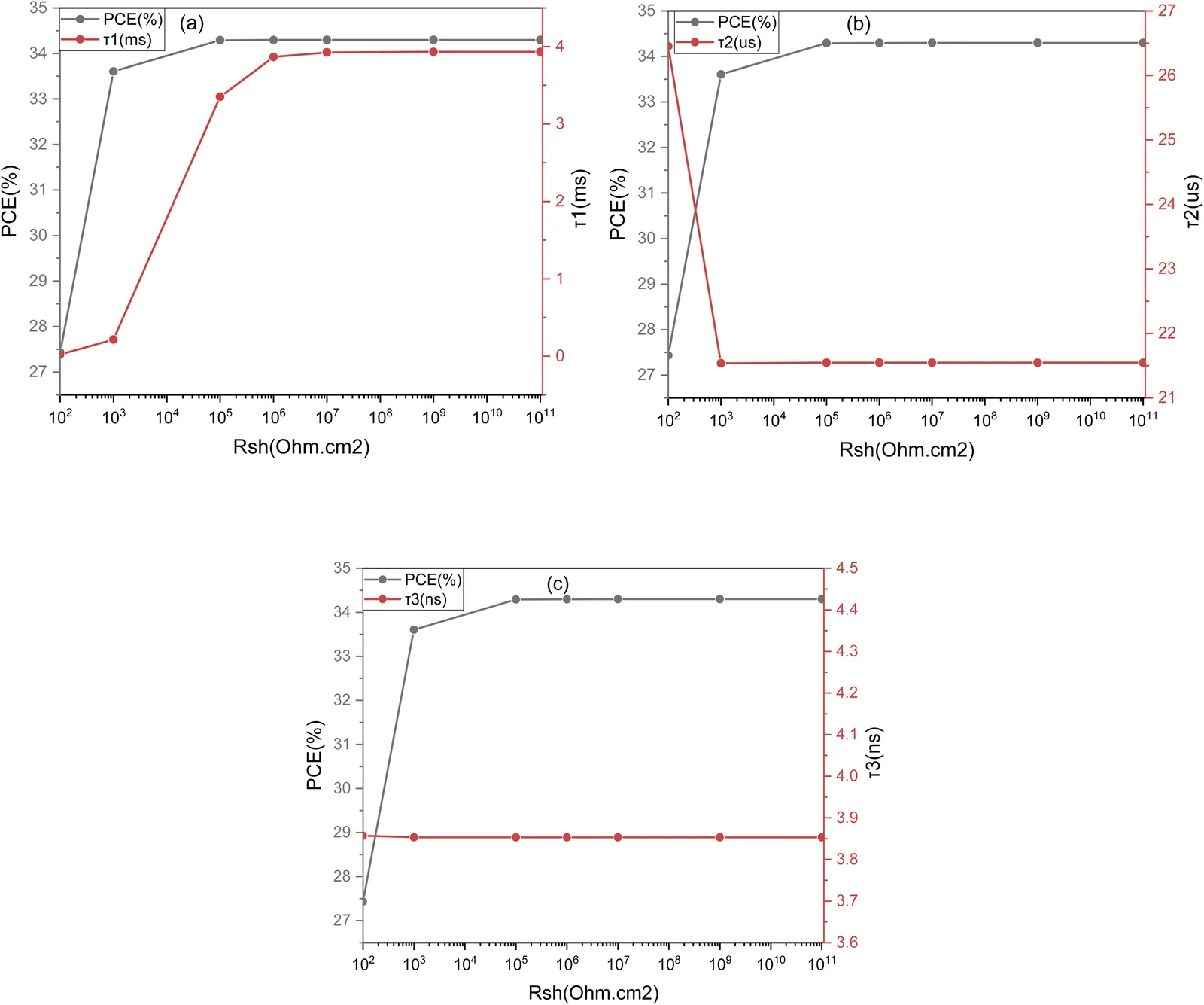
Effect of shunt resistance on PCE and relaxation times: (a) *τ*_1_, (b) *τ*_2_, and (c) *τ*_3_.

## 4 Conclusion

The electrical behavior of the ITO/ZnSe/CuInSe_2_/WSe_2_/Cu-doped carbon solar cell was investigated using DC and small-signal AC simulations in SCAPS-1D. Among the investigated absorber thicknesses, 1.2 µm yielded the highest simulated efficiency, approximately 33.4%, reflecting a balance between increasing photocurrent and decreasing *V*_oc_ and FF. A controlled work-function comparison showed that the adopted Cu-doped carbon contact approximately preserves the DC performance obtained with Au. Increasing series resistance primarily reduced FF and efficiency, whereas increasing shunt resistance improved performance by reducing parallel leakage. Disabling interface defect states produced small photovoltaic changes, with greater sensitivity to the ZnSe/CIS interface.Combined impedance and modulus analyses highlighted overlapping electrical contributions, represented using a three-branch RC equivalent circuit. Examining the fitted resistances and capacitances separately clarified the changes in their characteristic times. The parameter sensitivities support proposed contributions from recombination and parallel leakage at low frequencies, transport-related dynamics at intermediate frequencies, and the back-contact region at high frequencies. In particular, the controlled rear-contact work-function variation supports the latter assignment. These associations provide a physical interpretation of the fitted response, while the relaxation times alone do not establish the origin of the efficiency maximum. Together, DC analysis and frequency-dependent characterization provide complementary insight into the electrical response of the modeled CIS solar cell.

## Author contributions

Abdelhadi Mouhiha: conceptualization, methodology, software, validation, writing – original draft, formal analysis, investigation, resources, data curation, writing – review & editing, visualization. Abdelaziz Amine: conceptualization, methodology, software, writing – reviewing and editing. Reddad El Moznine: conceptualization, methodology, software, writing – reviewing and editing, supervision.

## Conflicts of interest

The authors declare that they have no conflict of interest.

## Data Availability

The data supporting the findings of this study are included within the article. Additional simulation data and SCAPS-1D output files are available from the corresponding author upon reasonable request.
